# Novel Pan-Coronavirus 3CL Protease Inhibitor MK-7845: Biological and Pharmacological Profiling

**DOI:** 10.3390/v16071158

**Published:** 2024-07-18

**Authors:** Nadine Alvarez, Gregory C. Adam, John A. Howe, Vijeta Sharma, Matthew D. Zimmerman, Enriko Dolgov, Risha Rasheed, Fatima Nizar, Khushboo Sahay, Andrew M. Nelson, Steven Park, Xiaoyan Zhou, Christine Burlein, John F. Fay, Daniel V. Iwamoto, Carolyn M. Bahnck-Teets, Krista L. Getty, Shih Lin Goh, Imad Salhab, Keith Smith, Christopher W. Boyce, Tamara D. Cabalu, Nicholas Murgolo, Nicholas G. Fox, Todd W. Mayhood, Valerie W. Shurtleff, Mark E. Layton, Craig A. Parish, John A. McCauley, David B. Olsen, David S. Perlin

**Affiliations:** 1 Center for Discovery and Innovation, Hackensack Meridian Health, 111 Ideation Way, Nutley, NJ 07110, USAdavid.perlin@hmh-cdi.org (D.S.P.); 2 Merck & Co., Inc., Rahway, NJ 07065, USA

**Keywords:** coronavirus, SARS-CoV-2, MERS-CoV, 3CLPro, antivirals, protease inhibitors

## Abstract

Severe acute respiratory syndrome coronavirus type 2 (SARS-CoV-2) continues to be a global threat due to its ability to evolve and generate new subvariants, leading to new waves of infection. Additionally, other coronaviruses like Middle East respiratory syndrome coronavirus (MERS-CoV, formerly known as hCoV-EMC), which first emerged in 2012, persist and continue to present a threat of severe illness to humans. The continued identification of novel coronaviruses, coupled with the potential for genetic recombination between different strains, raises the possibility of new coronavirus clades of global concern emerging. As a result, there is a pressing need for pan-CoV therapeutic drugs and vaccines. After the extensive optimization of an HCV protease inhibitor screening hit, a novel 3CLPro inhibitor (MK-7845) was discovered and subsequently profiled. MK-7845 exhibited nanomolar in vitro potency with broad spectrum activity against a panel of clinical SARS-CoV-2 subvariants and MERS-CoV. Furthermore, when administered orally, MK-7845 demonstrated a notable reduction in viral burdens by >6 log orders in the lungs of transgenic mice infected with SARS-CoV-2 (K18-hACE2 mice) and MERS-CoV (K18-hDDP4 mice).

## 1. Introduction

The discovery of new antiviral drugs is imperative in combating coronavirus infections, considering the burden of endemic and pandemic strains on human health. The available SARS-CoV-2 vaccines have successfully contributed to the controlling of the COVID-19 pandemic. Yet, the high frequency of SARS-CoV-2 mutations and the continued emergence of new sub-variants that evade or limit protection from antibodies and vaccines represent a challenge for the controlling of future waves of infections. This highlights the current and ongoing need for effective antiviral agents. Regarding MERS-CoV, a 2022 global summary from the WHO (World Health Organization) indicated a strong reduction in the number of cases reported since the beginning of the COVID-19 pandemic, which may the reflect prioritization of epidemiological surveillance for COVID-19. It is important to note that MERS-CoV infection has a higher mortality rate compared to COVID-19 and, currently, no commercial vaccines are available [1]. Among the targets for antiviral therapy against coronaviruses, including SARS-CoV-2, the main 3-chymotrypsin-like protease (Mpro, also referred to as 3CLPro) represents one of the most attractive given its pivotal role in viral replication [2], its homology among coronaviruses [3], and its recognition of a unique cleavage sequence not utilized by human proteases [4]. Early in the pandemic, numerous programs were established to identify novel inhibitors which target 3CLPro [5].

The inhibitor nirmatrelvir (PF-07321332) emerged as the most salient example of this class, and acts by forming a reversible covalent bond between its nitrile warhead and the catalytic cysteine (Cys145) residue of 3CLPro. Nirmatrelvir is orally bioavailable and, as part of Paxlovid, is co-administered with the potent CYP3A4 inhibitor ritonavir as a pharmacokinetic boosting agent, which increases nirmatrelvir concentrations to the target exposure level. Paxlovid was approved for use in newly diagnosed patients to reduce COVID-19-related hospitalizations [6]. However, the use of ritonavir, which increases the plasma levels of nirmatrelvir by up to 20-fold [7], has the limitation that it can cause hepatic impairment and modulates the metabolism of many drugs. Another selective SARS-CoV-2 3CLPro inhibitor, ALG-097111, showed potent activity against SARS-CoV-2 in vitro replication as well as the efficient inhibition of viral replication in the lungs of infected hamsters [8].

Boceprevir, a ketoamide-bearing antiviral used clinically in the treatment of the hepatitis C virus (HCV) via covalent inhibition of the NS3/4A serine protease, was reported to inhibit the 3CLPro of SARS-CoV-2 [9]. Although boceprevir only demonstrated weak cellular antiviral activity against SARS-CoV-2 [10], it shows attractive pharmacologic properties that enable good bioavailability and oral dosing (PO) [11]. Recently, a novel reversible covalent protease inhibitor from the ketoamide class, MK-7845, has been identified. The compound incorporates a unique P1 difluoroalkyl group, replacing the γ-lactam present in most covalent 3CLPro inhibitors reported to date [12]. MK-7845 exhibits favorable pharmacokinetics without boosting and physicochemical properties that provide good oral bioavailability and improved antiviral activity. The biochemical activity of the compound was characterized against a panel of recombinant enzymes, including wild-type SARS-CoV-2 3CLPro as well as other 3CLPro enzymes from different members of the Coronaviridae family [12]. In this study, we further characterized the inhibitory activity of MK-7845 against SARS-CoV-2 and MERS-CoV infection, utilizing both in vitro viral inhibition assays and in vivo animal models for efficacy. Our results demonstrate the broad-spectrum activity of MK-7845 against coronaviruses and support the potential for a treatment for SARS-CoV-2 and future coronavirus outbreaks.

## 2. Materials and Methods

### 2.1. Compounds, Cell Lines and Virus Strains

MK-7845 was obtained from Merck & Co., Inc., Rahway, NJ, USA; remdesivir (GS-5734) and nirmatrelvir (PF-07321332), used as comparators for the in vitro and/or in vivo assays, were purchased from Selleckchem, Houston TX, USA, Cat. No. S8932 and Aobious, Gloucester, MA, USA, Cat. No. AOB14800, respectively.

Human Huh7 cells were grown in culture medium high-glucose Dulbecco’s Modified Eagle’s Medium (DMEM) glutaMAX-1 containing 10% heat-inactivated fetal bovine serum (FBS), non-essential amino acids (NEAA), and 1-% penicillin-streptomycin (Pen-Strep, Gibco ThermoFisher, Allentown, PA, USA, Cat. No. 10378016). Human A549 cells (ATCC, Manassas, VA, USA, Cat. No. CCL-185) were cultured in high-glucose DMEM (ATCC, Manassas, VA, USA, Cat. No. 30-2002) supplemented with L-glutamine and sodium pyruvate. African green monkey kidney cells (Vero E6+TMPRSS2) were obtained from XenoTech, Japan (JCRB1819, Lot No. 2222020) and maintained in high-glucose DMEM, supplemented with 10% FBS (Thomas Scientific, Manassas, VA, USA, Cat. No. C788U22) and 1% Pen-Strep. All cell lines were sub-cultured and maintained at 37 °C with 5% CO_2_ and 90% relative humidity.

The following SARS-CoV-2 strains, and the MERS-CoV strain, were obtained through the Biodefense and Emerging Infections Research Resources repository (BEI Resources, Manassas, VA, USA):

- SARS-CoV-2, USA-WA1/2020 (Cat. No. NR-53873, Lot No. 70039812);
- SARS-CoV-2 Lineage B.1.1.529, Omicron Variant (Cat. No. NR-56461, Lot No. 70049434);
- SARS-CoV-2 Lineage BA.2; Omicron Variant (Cat. No. NR-56520, Lot No. 70051592);
- SARS-CoV-2 Lineage BA.5; Omicron Variant (Cat. No. NR-56798, Lot No. 70053884);
- SARS-CoV-2 Lineage BQ.1; Omicron Variant (Cat. No. NR-58975, Lot No. 70057300);
- SARS-CoV-2 Lineage BF.5; Omicron Variant (Cat. No. NR-58716, Lot No. 70055929);
- MERS-CoV icMERS-CoV-RFP-ΔORF5 (Cat. No. NR-48813, Lot No. 63140603);
- MERS-CoV EMC 2012 (Cat. No. NR-44260, Lot No. 70002733).

Three additional SARS-CoV-2 Omicron subvariants were recovered from a nasopharyngeal swab taken from patients, as part of the NJDOH surveillance program for COVID-19, and confirmed by whole-genome sequencing using the service of the NY Genome Center:

- SARS-CoV-2 sub-lineage XBB.1.5, Omicron Variant (CVD357), EPI_ISL_16346196;
- SARS-CoV-2 sub-lineage XBB.1.16.1, Omicron Variant (CVD372), EPI_ISL_17617817;
- SARS-CoV-2 sub-lineage EG.5.1, Omicron Variant (CVD385), EPI_ISL_18107634;
- SARS-CoV-2 sub-lineage BA.2.86.1.1, Omicron Variant JN.1 (CVD408), EPI_ISL_18669842.

All the virus strains were propagated in Vero E6+TMPRSS2 cells using high-glucose DMEM supplemented with 10% fetal bovine serum (FBS, Thomas Scientific Cat. No. C788U22) and 1% Pen-Strep. The virus titer was determined by the 50% tissue culture infective dose (TCID_50_), calculated according to the Reed–Muench method [13], and the PFU assays were performed by using a liquid overlay and a fixation-staining method to enumerate the PFU per mL [14].

### 2.2. Enzymatic Activity Assays

The enzymatic activity of recombinantly expressed 3CLPro enzymes from different coronaviruses was measured using the following synthetic quenched FRET peptide: CP488-ESATLQSGLRKAK- (CPQ2)-NH2 (CPC Scientific, San Jose, CA, USA). The substrate functions as a generic peptide substrate for 3CLPro enzymes and is designed based on the nsp4/nsp5 cleavage sequence [15]. Recombinant protein was generated by transformation and expression in *E. coli* BL21-Gold (DE3) competent cells for the plasmid containing 3CLPro (1-306) from SARS-CoV-2 in the pGEX-6p-1 Vector. The plasmid has an N-terminal self-cleavage recognition site (AVLQ) that naturally cleaves off GST-tag during expression and a modified Prescission Protease site at C-terminus to cleave off His-tag. Following lysis, the protein was purified by IMAC. His-GST-Prescission Protease (Cytiva, Marlborough, MA, USA), then added overnight at 4 °C to cleave the His-tag while in dialysis to remove any imidazole from the buffer. A GST-column was used to remove His-GST-Prescission Protease and any protein for which self-cleavage was not successful followed by reverse IMAC to remove the His-tag. Final purification involved a gel-filtration S200 column in a buffer containing 50 mM HEPES pH 7.3, 150 mM NaCl, 5% glycerol, and 1 mM tris(2-carboxyethyl) phosphine (TCEP).

To measure the enzymatic activity, compounds in DMSO were acoustically pre-dispensed in black 384-well ProxiPlates (Perkin Elmer, Shelton, CT, USA) in a 10-point 3-fold dilution followed by addition of SARS-CoV-2 3CLPro wild-type (5 nM final) in 50 mM HEPES, pH 7.5, 0.01% Triton X-100, 0.01% BSA, and 2 mM DTT. After pre-incubation for 30 min at 25 °C, the reaction was initiated with the addition of the peptide substrate (15 µM final) and incubated for 4 h at 25 °C, followed by quenching with a high-dose inhibitor. The fluorescence of the cleaved substrate was read on a Pherastar MultiLabel Reader (BMG Labtech, Cary, NC, USA) at Ex495, Em520.

**Figure 1:**
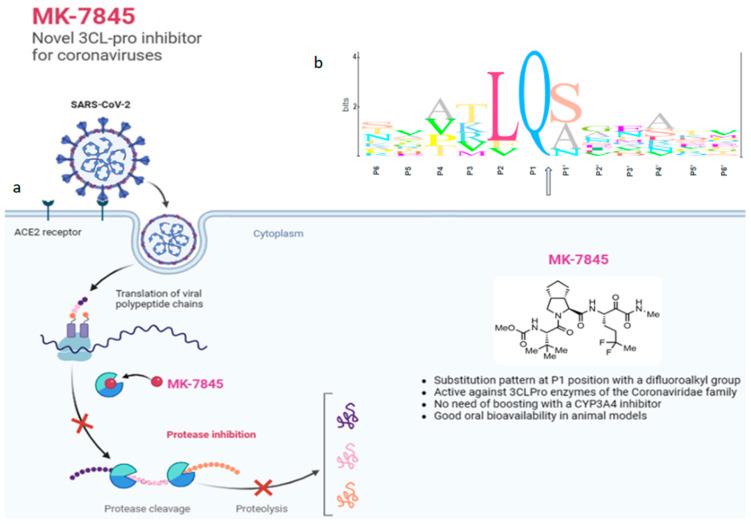
(**a**) Structure and mechanism of action of MK-7845; (**b**) Specific recognition sequences by 3CL proteases in coronaviruses, where the arrow indicates the 3CL cysteine protease cleavage site. Adapted from “Coronavirus Replication Cycle”, by BioRender.com (2020), accessed on 26 January 2024. Retrieved from https://app.biorender.com/biorender-templates.

For the logo plot of NSP5 processing sites (Figure 1b), one hundred and eighty two representative SARS-CoV2 sequences, as identified from nextstrain.org, were pulled from GISAID (EPI_SET_240702sw). NSP5 processing sites for NSP4-NSP16 were aligned for the sequences and a logo plot was generated using Geneious Prime v. 2023.0.3. See Supplementary Table S1.

### 2.3. Replicon-Based Screening Assay for SARS-CoV-2 and MERS-CoV

The antiviral potency of compounds in the replicon-based assay was determined as described [12]. Briefly, compounds were dispensed into 384-well poly-D-lysine-coated microplates (Corning, Glendale, AZ, USA, Cat. No. 356663) at 0.2 µL/well, and dissolved in DMSO via an ECHO acoustic dispenser (Labcyte, San Jose, CA, USA). Each compound was diluted into a 10-point, 3-fold titration series, with final concentrations from ~42,000 nM to 2 nM. DMSO-only and an internal 3CLPro inhibitor were also dispensed into designated wells as minimum and maximum inhibition effect controls, respectively. A549 cells were electroporated with the SARS-CoV-2 replicon RNA and dispensed into each well (10,000 cells in 50 µL/well) using a Bravo automated liquid dispenser (Agilent, Santa Clara, CA, USA). Cells were incubated with compounds at 37 °C with 5% CO_2_ and 90% relative humidity. At ~40 h post-treatment, the number of green cells per well was evaluated using an Acumen eX3 scanner (SPT Labtech, Melbourn, UK).

### 2.4. hCoV-229E and hCoV-OC43 Antiviral Cytopathic Effect Assay

The antiviral activity against hCoV-229E and hCoV-OC43 variants was evaluated by assessing the ability of inhibitors to prevent a viral-induced cytopathic effect (CPE) in Huh7 cells. Three days prior to the assay, 3 × 10^6^ cells per T175 flask were prepared in culture media. On the day of assay, medium was aspirated, and cells were washed with Dulbecco’s phosphate-buffered saline (Gibco, ThermoFisher, Fair Lawn, NJ, USA, Cat. No. 14190144) and detached with 3 mL of Trypsin-EDTA (Gibco ThermoFisher, Fair Lawn, NJ, USA, Cat. No. T4174). Cells were diluted in 10 mL of assay media (DMEM, 4% FBS, NEAA, 1% Pen-Strep), counted, and diluted further in assay media to 8 × 10^4^ cells/mL.

Compound titrations on 384-well white tissue culture-treated assay plates (Corning, Glendale, AZ, USA, Cat. No. 3570) were prepared in a 10-point, 3-fold titration series. Cells were then dispensed into conical tubes for bulk infection with hCoV-229E (ATCC, Manassas, VA, USA, Cat. No. VR-740) or hCoV-OC43 (ATCC, Manassas, VA, USA, Cat. No. VR-1558D) variant at a target multiplicity of infection (MOI) of 0.04. After adding the virus, the cells were then immediately dispensed into assay plates at 25 µL/well in columns 3–24 and uninfected cells added to columns 1–2. The cells were incubated at 35 °C with 5% CO_2_ and 90% relative humidity. After 120 h, cell viability was assessed using the CellTiter-Glo^®^ 2.0 kit (Promega, Madison, WI, USA) following manufacturer’s protocol. Luminescent signals were read using an Envision multimode plate reader (PerkinElmer, Shelton, CT, USA). All raw data were normalized to percent CPE using respective minimum and maximum effect controls on each assay plate. The normalized data were plotted as a dose–response curve, and half-maximal effective concentration (EC_50_) was determined from nonlinear four-parameter curve fitting using IDBS ActivityBase version 9.7.1. To monitor compound toxicity, compound was added to a second assay plate with 25 µL of uninfected cells added to each well. After 120 h, cell viability was assessed using the CellTiter-Glo^®^ 2.0 kit (Promega, Madison, WI, USA, Cat. No. G9241).

### 2.5. Antiviral and Toxicity Assays Using Live SARS-CoV-2 and MERS-CoV Viruses

MK-7845 in vitro activity was evaluated against SARS-CoV-2 and MERS-CoV, using medium throughput whole cell assays. Amounts of 3 × 10^3^ Vero E6+TMPRSS2 cells per well were seeded in 384-well plates using an EL406 automated liquid dispenser (Agilent, Santa Clara, CA, USA) and cultured for up to 24 h at 37 °C and 5% CO_2_. MK-7845 and the reference drugs (remdesivir and nirmatrelvir) were dispensed into the cells via a D300e digital dispenser (Tecan, Medford, MA, USA) using 10 mM stocks in 100% DMSO. To evaluate the half-maximal inhibitory (IC_50_) and EC_50_ of the compounds, the cells were pretreated for 2 h with 3-fold serial dilutions and final drug concentrations from 36,000 nM to 1.8 nM, using a maximum limit of 0.5% DMSO/well. After dispensing the compounds, cells were additionally pre-treated with the P-glycoprotein (P-GP) inhibitor at 2000 nM/well (elacridar, GF120918 MedChem Express, Monmouth Junction, NJ, USA, Cat. No. HY-50879). Following 2 h of incubation, a single-use frozen stock of each virus strain was diluted in DMEM and added to the wells at MOI of 0.5 or MOI 0.1 (in the case of EG.5.1, XBB.1.16.1, and JN.1). Plates were incubated at 37 °C and 5% CO_2_ for 48 h (to evaluate virus growth through fluorescence detection) and additional plates were incubated at 72 h (to determine cell viability and compound toxicity by luminescence detection).

#### 2.5.1. Fluorescence Detection for Inhibitory Concentration

The media in the wells were removed and the plates were fixed with 25 µL of 10% neutral buffered formalin (NBF) for 24 h at 4 °C. Plates from the SARS-CoV-2 assay were washed three times with PBS and incubated with SARS-CoV-2 nucleocapsid recombinant protein antibody (BioLegend, San Diego, CA, USA, Cat. No. 940902, dilution 1:5000), followed by incubation with a goat anti-mouse IgG (H+L) cross-adsorbed secondary antibody conjugated to Alexa Fluor™488 (Invitrogen, Fair Lawn, NJ, USA, Cat. No. A-11001, dilution 1:10,000). A final staining step was performed using Hoechst 33342 dye (ThermoFisher, Fair Lawn, NJ, USA, Cat. No. 62249) to counterstain the nucleus at a final concentration of 0.1%. Plates from the assay using the MERS-CoV reporter virus expressing a red fluorescent protein (RFP) were directly washed three times with PBS 1× and stained with Hoechst as described above. The virus growth was analyzed by high-content imaging (HCI) in a Cytation 10 confocal imaging reader (Agilent, Santa Clara, CA, USA), using the green-fluorescent channel for SARS-CoV-2 and the red-fluorescent channel for MERS-CoV. The percentage of infected cells was used to determine the IC50 activity.

#### 2.5.2. Luminescence Detection for Effective and Cytotoxic Concentration

The half-maximal cytotoxic concentration (CC_50_) was assessed in Vero E6+TMPRSS2 cells using two-fold serial dilutions of the compounds, with a concentration ranging from 100,000 nM to 780 nM. For the CPE, EC_50_, and CC_50_ evaluations, the plates were incubated at 37 °C and 5% CO_2_ for 72 h. The CPE was evaluated under a phase contrast microscope and the cell viability and toxicity of the compounds were measured using the Promega CellTiter-Glo^®^ 2.0 Cell Viability Assay (Promega, Madison, WI, USA, Cat. No. G9241). The EC_50_ was calculated based on the viral CPE inhibition and the relative luminescence units (RLU) were used to correlate the cell viability and evaluate the toxicity of the compounds in the cells (CC_50_).

#### 2.5.3. Quality Assessment of In Vitro Assays

The reproducibility of the compounds’ internal replicates as well as the performance of the controls in the inter-plate and inter-day tests (Z’ factor) were determined. The Z’ factor values in all the assays exceed 0.5, with an average of 0.7 ± 0.1, the signal/noise (S/N) ratio was close to - or >3-fold and the coefficient of variation (CV) was ≤20, indicating an acceptable assay performance as established in a comparative study [16].

### 2.6. Pharmacokinetic Studies

CD-1 female mice (22–25 g) were used in oral pharmacokinetic (PK) studies. All animal studies were ethically reviewed and carried out in accordance with European Directive 2010/63/EU and the GSK Policy on the Care, Welfare and Treatment of Animals. MK-7845 intravenous (IV) dosing at 5 mg/kg was formulated in 30% Captisol. MK-7845 PO doses of 100 mg/kg, 250 mg/kg, 500 mg/kg, or 1000 mg/kg were formulated in 10% Tween 80. Aliquots of 50 μL of blood were taken by puncture of the lateral tail vein from each mouse (*n* = 3 per route and dose) at 30 min, 2 h, 4 h, 7 h, and 11 h after the initial dose for oral dosing and at 5 min, 0.25 h, 1 h, 3 h, 5 h, 7 h, and 24 h for IV dosing. Blood for therapeutic drug monitoring from MERS-CoV-infected mice was collected 12 h after the final dose by cardiac stick. Blood was captured in CB300 blood collection tubes containing K2 EDTA and stored on ice. Plasma was recovered after centrifugation and stored at −80 °C until analyzed by high-pressure liquid chromatography coupled to tandem mass spectrometry. Lungs were placed in microcentrifuge tubes and stored at −80 °C. PK parameters were determined using non-compartmental PK analysis methods supported by the Excel PK solver Add-in. Area under the curve values were determined using trapezoidal integration.

### 2.7. LC-MS/MS Analytical Methods

Neat 1 mg/mL DMSO stock of MK-7845 was serial diluted in 50/50 Acetonitrile (ACN)/Milli-Q water to create standard curve solutions. Standards were created by adding 10 µL of spiking solutions to 90 µL of drug-free plasma (CD-1 K2 EDTA Mouse, Bioreclamation IVT, Hicksville, NY, USA), or homogenate made using in-house-collected drug-free CD-1 mouse lungs. Mouse lung tissues and drug-free control mouse lung were weighed and homogenized in 4 volumes of PBS to a final 5x dilution factor. Homogenization was achieved using a FastPrep-24 instrument (MP Biomedicals, Solon, OH, USA) and 1.4 mm zirconium oxide beads (Bertin Corp., Rockville, MD, USA). Ten microliters of control, standard, or study biological matrix were added to 100 µL of ACN protein precipitation solvent containing 10 ng/mL of the internal standards Verapamil (Sigma Aldrich, Saint Louis, MO, USA). Extracts were vortexed for 5 min and centrifuged at 4000 RPM for 5 min. Seventy-five microliters of supernatant were transferred for HPLC-MS/MS analysis and diluted with 75 µL of Milli-Q deionized water.

LC-MS/MS analysis was performed on a Sciex Applied Biosystems Qtrap 6500+ triple-quadrupole mass spectrometer coupled to a Shimadzu LC-30AD Nexera X2 UHPLC system (Nanuet, NY, USA) to quantify each drug in plasma. Chromatography was performed on an Agilent SB-C8, Santa Clara, CA, USA (2.1 × 30 mm; particle size, 3.5 µm) using a reverse phase gradient. Milli-Q deionized water with 0.1% formic acid was used for the aqueous mobile phase and 0.1% formic acid in ACN was used for the organic mobile phase. Multiple-reaction monitoring of parent/daughter transitions in electrospray positive-ionization mode was used to quantify all analytes. Quantification was performed by integration of the combined epimers as previously detailed [12]. The following multiple-reaction monitoring (MRM) transitions were used for MK-7845 (517.30/346.10) and Verapamil (455.40/165.00). Sample analysis was accepted if the concentrations of the quality control samples were within 20% of the nominal concentration. Data processing was performed using Analyst software (Applied Biosystems Sciex, version 1.6.2).

### 2.8. In Vivo Efficacy Models: Ethics, Biosafety and Study Design

All animal experiments were approved by the Institutional Animal Care and Use Committee (IACUC) at Hackensack Meridian Health, according to guidelines outlined by the Association for the Assessment and Accreditation of Laboratory Animal Care and the U.S. Department of Agriculture. The in vivo efficacy of MK-7845 was evaluated in transgenic K18-hACE2 mouse model expressing the ACE2 for SARS-CoV-2 infection, and transgenic K18-hDPP4 mouse model expressing the human dipeptidyl peptidase 4 (DPP4) for MERS-CoV infection [17,18]. The animals used in the studies were purchased from Jackson Laboratories and housed at the Center for Discovery and Innovation (CDI) Research Animal Facility. The animals were kept in individually ventilated caging units and were handled under sterile conditions for a minimum of 72 h for acclimation prior to study initiation.

Nirmatrelvir has previously been utilized as a positive control for reducing lung viral burden in a transgenic K18-hACE2 mouse model of SARS-CoV-2 infection [19,20]. However, to the best of our knowledge, this is the first instance where nirmatrelvir is included as a comparator for the protection against MERS-CoV infection in the transgenic K18-hDPP4 mouse model. To validate the antiviral efficacy of nirmatrelvir in this in vivo model, we evaluated an orally delivered BID therapy at 300 mg/kg and 1000 mg/kg and assessed the efficacy with administration beginning 1 h prior to infection with SARS-CoV-2 or MERS-CoV. As a result, both infection models treated with 1000 mg/kg nirmatrelvir showed significant reduction in the lung viral loads when dosed for 3 days post-infection (dpi), and this effect was more significant in the K18-hDPP4 mice’s protection against MERS-CoV (Figure S1). According to these results, we decided to use nirmatrelvir 1000 mg/kg as a comparator for both SARS-CoV-2 and MERS-CoV animal models.

### 2.9. Antiviral Efficacy Evaluation of MK-7845 in Prophylactic and Therapeutic Mode in SARS-CoV-2 and MERS-CoV Infection Mice Models

MK-7845 was evaluated for both prophylactic and therapeutic antiviral efficacy against coronaviruses SARS-CoV-2 and MERS-CoV, with nirmatrelvir serving as a positive control. Nirmatrelvir previously showed high in vitro potency with IC_50_/EC_50_ = 100 nM–300 nM. MK-7845 was administered 1 h prior to infection (100 mg/kg, 250 mg/kg, and 500 mg/kg), 4 h post-infection (hpi) (100 mg/kg, 250 mg/kg, and 500 mg/kg) and 12 hpi (500 mg/kg), while nirmatrelvir 1000 mg/kg was administered 4 h post-infection.

Male and female transgenic K18-hACE2 mice (8–10 weeks; *n* = 5–6 per group) were intranasally challenged with SARS-CoV-2 virus strain USA-WA1/2020. The virus inoculum contained 50 µL of virus stock with a final infection dose of 4.6 × 10^4^ TCID_50_. Animals were administered MK-7845 or vehicle (10% Tween 80 in 10 mM sodium citrate buffer, pH 4) via oral gavage 1 h prior to infection at doses of 100 mg/kg, 250 mg/kg, and 500 mg/kg, whereas post-infection administration started at 4 hpi at doses of 100 mg/kg, 250 mg/kg, and 500 mg/kg and 12 hpi at a dose of 500 mg/kg.

Similarly, the male and female transgenic K18-hDPP4 mice (7–9 weeks; *n* = 6 per group) were intranasally challenged with MERS-CoV virus strain EMC 2012. The virus inoculum contained 50 µL of virus stock with a final infection dose of 8.0 × 10^4^ TCID_50_. Animals were administered MK-7845 or vehicle (10% Tween 80 in 10 mM sodium citrate buffer, pH 4) via oral gavage 1 h prior to infection and at 200 mg/kg, 500 mg/kg, and 1000 mg/kg, whereas post-infection administration started at 4 hpi at doses of 200 mg/kg, 500 mg/kg, and 1000 mg/kg and 12 hpi at a dose of 1000 mg/kg.

In the SARS-CoV-2 and MERS-CoV models, nirmatrelvir (*n* = 6) was administered at a dose of 1000 mg/kg, starting at 4 hpi, serving as a comparator. Following infection, mice were treated twice a day for 3 days, and body weights were recorded. On day 4, mice were euthanized by carbon dioxide inhalation, and blood and lung tissues were collected. The left half of the lung was homogenized for viral burden determination whereas the right half of the lung was inflated and fixed in 10% NBF for histopathology analysis. The samples were maintained in the fixation solution for a minimum of 24 h before being safely removed from the ABSL-3 and sent for histopathology analysis. For the non-terminal blood collection, mice were manually restrained, and blood was collected (~0.05 mL) into K2 EDTA Microvette^®^ tubes by submandibular puncture, using a 25 G needle or disposable lancet. Terminal blood collection was performed by cardiac puncture. The blood was centrifuged for 15 min at 3000 RPM (1500× *g*) at 4 °C and plasma was carefully transferred using a pipette with aerosol barrier tip into a new 1.5 mL screw cap tube and stored at −80 °C until PK analysis.

#### 2.9.1. Viral Load Determination in Lungs

Viral burdens in lungs were evaluated by TCID_50_ assay, PFU assay, and RT-qPCR. The left half of each lung was weighed and homogenized in an OCTOMACS tube containing 2.5 mL of DMEM + 2% FBS + 1% Pen-Strep. Homogenates were centrifuged and clear supernatants were collected.

#### 2.9.2. TCID_50_ Assay

A 72-h cytopathic assay was performed in Vero E6+TMPRSS2 cells using Promega Cell Titer-Glo^®^ 2.0 Cell Viability Assay with lung homogenates. The lower limit of quantification (LLOQ) for the TCID_50_ assay was 2.4 log TCID_50_ per gram of tissue. The CPE induced by the virus was additionally evaluated by visual inspection of the cell monolayer integrity under brightfield at 10× magnification.

#### 2.9.3. PFU Assay

One hundred microliters of the clarified homogenate resuspended in DMEM were directly added to 24-well plates containing 2.5 × 10^5^ Vero E6+TMPRSS2 cells/well or 10-fold serial dilutions up to 10^−6^ were prepared in DMEM then added to wells. After inoculation, the 24-well plates were gently rocked for 1 h at 37 °C. Then, 500 µL of a pre-warmed overlay mixture (2× MEM + 2.5% Cellulose (1:1)) were added and plates were incubated for 72 h at 37 °C. The media were removed, and plates were fixed with 10% NBF, stained with 0.5% crystal violet, and washed with water and enumerated. For the PFU assay, the LLOQ was ~250 PFU per gram of lung tissue.

#### 2.9.4. RNA Extraction and RT-PCR

Mice lung homogenates (225 µL) were inactivated with proteinase K (25 µL) for 1 h at 65 °C. The viral RNA was isolated from the samples, using the Qiagen QIAcube HT (Germantow, MD, USA) automated mid-to-high-throughput nucleic acid purification instrument with the QIAamp 96 Virus QIAcube HT Kit. For SARS-CoV-2, RT-qPCR was performed on samples using the E gene primer, probe panel, and RNase P gene as described before [21]. For MERS-CoV, the RT-qPCR was performed on the samples using the ORF1a gene for MERS-CoV and RNase P gene as the amplification control. The primer and probe sequences for SARS-CoV-2 and MERS-CoV are listed in Table S2a and Table S2b, respectively. The LLOQ for the RT-qPCR assay was 10,000 copies per gram of lung tissue.

#### 2.9.5. Histopathology Analysis

The lung tissue samples were sent to Histowiz Inc. (Queens, NY, USA) for further processing. These samples from SARS-CoV-2-infected K18-hACE2 mice and MERS-CoV-infected K18-hDPP4 mice were initially received in 10% NBF. Upon receipt, the lung samples were grossly inspected, processed, and paraffin embedded. Subsequently, sections were cut from the paraffin block, placed on glass slides, and stained with hematoxylin and eosin (H&E). The digitally scanned images of the H&E-stained lung sections were generated using the Aperio AT2 scanner from Leica Biosystems, Inc. (Buffalo Grove, IL, USA), and images were then uploaded onto a cloud platform for evaluation by a board-certified pathologist. For SARS-CoV-2-infected samples, the histopathology was evaluated for perivascular inflammation, bronchiolar epithelial cell degeneration or necrosis, peribronchial inflammation, intra-alveolar inflammation, and fibrinoid degeneration of the vascular wall [20,22]. A 4-point scoring system for assessment of epithelial degeneration/necrosis and inflammation was utilized: 0) within normal limits normal or absent; (1) localized, 33% lung field, mild severity; (2) multifocal, 34–66% of lung fields, with moderate severity; and (3) >67% of lung fields, marked severity of pathology (Table S3). A cumulative pathology score was calculated for each mouse by adding individual histopathological scores, and the average score was calculated per group.

In the case of MERS-CoV, lung pathology was evaluated as perivascular edema, thrombosis, cell debris in lymphatics, and cellular inflammation [17]. Parameters of consolidation (cellular inflammation, atelectasis, etc.) and edema were scored on a distribution approach: (1) normal/absent; (2) localized, 1–33% lung fields; (3) multifocal, 34–66% of lung fields; and (4) common, >67% of lung fields. Parameters of vascular thrombi and cell debris in lymphatics were scored on an incidence approach: (1) normal/absent; (2) <1 per 100× fields; (3) 1–2 per 100× fields; and (4) >2 per 100× fields (Table S4).

### 2.10. Statistical Analysis

All data was analyzed using GraphPad Prism (version 10.0.0) software package. The raw data obtained from the antiviral screening of compounds using the replicon-based and the whole cell-based assays were normalized to the untreated/uninfected and untreated/infected controls. The normalized data were plotted as inhibitory response versus concentration, and the IC_50_, EC_50_, and CC_50_ values were determined using a non-linear regression (curve fit), applying the equation for inhibitor vs. response variable slope (four parameters) and a 95% confidence interval. The EC_90_ response was estimated by using the EC_50_ values and the hillslope (H) information in the following equation:

*EC*_90_ = (90/(100 − 90)) ^√H*(*EC*_50_)

For TCID_50_, PFU assay, and RT-PCR, samples with a value below the LLOQ were assigned a value of 1/2 LLOQ for statistical analyses (1.2 LLOQ for TCID_50_ and PFU assay and 2 LLOQ for RT-PCR). The identified outliers were excluded from the statistical analysis. To evaluate the statistical significance between several groups from the in vivo studies, one-way ordinary ANOVA was used with Dunnett’s multiple comparisons post hoc test.

## 3. Results

MK-7845 is a novel 3CLPro inhibitor recently derived from extensive structure-based optimization of the P1-P4 substituents of a peptidomimetic scaffold [12], where the main objective was to improve its potency, minimize potential drug–drug interactions, and retain pharmacokinetics, enabling oral administration without boosting with ritonavir or other agents. One advantageous structural modification in the advanced compound MK-7845 included substituting the γ-lactam present at P1 of many 3CLPro inhibitors with a difluoroalkyl group, which is proposed to contribute to increased drug permeability (Figure 1a).

### 3.1. Characterization of the Biochemical and Cellular Spectrum of Coronavirus Activity of MK-7845

A panel of recombinant 3CLPro enzymes were expressed, purified, and evaluated for inhibition by MK-7845 utilizing a synthetic quenched fluorescence resonance energy transfer (FRET) substrate based on the nsp4/nsp5 cleavage sequence [15]. MK-7845 was identified to have potent activity against SARS-CoV-1 and SARS-CoV-2, with IC_50_ values of 19.2 nM and 8.7 nM, respectively. Activity was also observed biochemically versus MERS-CoV 3CLPro (IC_50_ = 318 nM); however, to support the activity, a 10–100-fold-higher enzyme concentration was required for the assay as compared to SARS-CoV-1 and SARS-CoV-2. In addition, MK-7845 was active against other human beta-coronavirus 3CLPro enzymes, hCoV-OC43 (IC_50_ = 17 nM), and hCoV-HKU1 (IC_50_ = 25.5 nM), as well as against the recombinant 3CLPro enzymes of the human endemic coronaviruses hCoV-229E (IC_50_ = 307 nM) and hCoV-NL63 (IC_50_ = 167 nM) (Table 1a).

To further characterize the interaction observed in co-crystal structures of MK-7845 with 3CLPro, wherein a nucleophilic attack of Cys145 on the reactive ketoamide forms a covalent adduct [12], differential scanning calorimetry studies were conducted. The compound was found to thermally stabilize the enzyme, as observed by a 21.8 °C shift from the apo enzyme (Figure S2a). The rapid recovery of the enzymatic activity upon a 100-fold dilution of MK-7845 from 10× IC_50_ to 0.1× IC_50_ indicates that the covalent interaction is reversible (Figure S2b).

The differential activity observed in the biochemical assays for hCoV-OC43 and hCoV-229E was confirmed in a CPE assay using Huh7 cells, wherein a 10-fold-higher inhibition was observed against hCoV-OC43 (EC_50_ = 370 nM) compared to hCoV-229E (EC_50_ = 3150 nM) (Table 1b). Considering these preliminary results, MK-7845 was further evaluated for cytotoxicity and cellular antiviral activity. A cell-based viral replication assay was performed using a non-infectious reporter replicon of SARS-CoV-2 in A549 cells wherein the EC_50_ value for MK-7845 was 15 nM (Table 1c). The observed potency was also similar in a MERS-CoV replicon assay in A549 cells (EC_50_ = 270 nM). These results were confirmed when using live SARS-CoV-2 and MERS-CoV viruses in a whole-cell-based assay, showing EC_50_ values of 140 nM and 390 nM, respectively.

For the additional characterization of MK-7845 against high-threat coronaviruses, a series of dose–response assays using live SARS-CoV-2 and MERS-CoV were performed. First, MK-7845 and the positive controls for inhibition (remdesivir and nirmatrelvir) were titrated in Vero E6+TMPRSS2 cells in the presence of the P-GP inhibitor elacridar for 72 h and CellTiter-Glo, to determine cell viability by the quantitation of ATP. For MK-7845 or nirmatrelvir, no effect on the cell viability was detected up to a maximum concentration of 100,000 nM (Figure S3). However, remdesivir showed moderate toxicity (CC_50_ = 20,000 nM).

Dose–response assays were performed against different SARS-CoV-2 Omicron subvariants and MERS-CoV in Vero E6+TMPRSS2 cells, in the presence of the P-GP inhibitor elacridar (Table 2). Following pretreatment with the compounds for 2 h, the cells were infected with a strain of SARS-CoV-2 or MERS-CoV. Forty-eight hours post-infection, MK-7845 reduced the viral growth, with IC_50_ values between 80 nM and 300 nM, when infected with different SARS-CoV-2 Omicron subvariants (Table 2; Figure S4). Similar in vitro potency was evidenced when the CPE was measured by CellTiter-Glo 72 h post-infection, with an EC_50_ between 90 nM and 330 nM (Table 2; Figure S5). Inhibition was also observed when treating MERS-CoV-infected cells with the compound, evidenced by IC_50_ and EC_50_ values of 360 nM and 460 nM, respectively (Table 2; Figure S6a,b). The EC_90_ values estimated using the EC_50_ and the hillslope (H) suggested 90% inhibition against both virus strains within the same range of concentrations.

The selectivity index (SI), which defines the window between the cytotoxicity and the antiviral activity, was determined for the three compounds by dividing the CC_50_ value by the EC_50_ value. Considering that the CC_50_ activity of MK-7845 is >100,000 nM (Figure S3), the in vitro selectivity index was determined to be >300–>1000 against SARS-CoV-2, and >216 against MERS-CoV (Table 2). Previously, it has been postulated that a SARS-CoV inhibitor with an SI of greater than 100 and an EC_50_ of not higher than 1000–3000 nM should be able to achieve sufficiently high plasma and tissue drug levels after systemic administration, and thus be effective in vivo [23].

### 3.2. Pharmacokinetics

**Figure 2:**
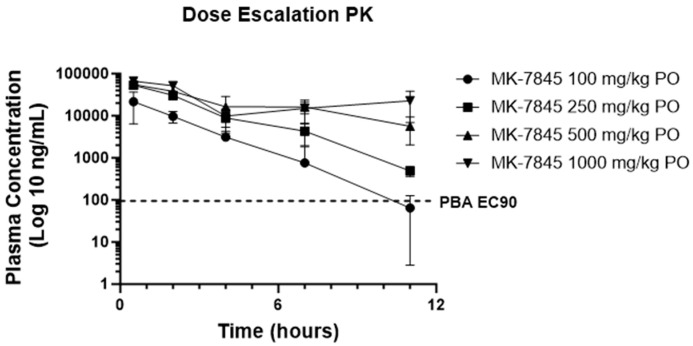
CD-1 mouse pharmacokinetic profile of MK-7845. Plasma concentration time profile of MK-7845 in CD-1 female mouse plasma following oral doses of 100 mg/kg, 250 mg/kg, 500 mg/kg, and 1000 mg/kg in 10% Tween 80. Mean and standard deviations are shown, *n* = 3 mice per dose level. PBA of SARS-CoV-2 replicon assay EC_90_ is represented by the dashed line.

The PO and IV PK profiling of MK-7845 was performed in uninfected CD-1 mice to determine the exposure profiles for SARS-CoV-2 and MERS-CoV efficacy studies. IV dosing of MK-7845 at 5 mg/kg demonstrated a terminal half-life of 0.79 h, a clearance of 17.5 mL/min/kg, and a volume of distribution of 1.2 L/kg. The PO profiling of MK-7845 in uninfected CD-1 mice using doses of 100 mg/kg, 250 mg/kg, 500 mg/kg, or 1000 mg/kg demonstrated areas under the curve (AUC) of 49.1, 146.1, 238, and 306 µg*h/mL for the respective dose levels. The total plasma concentrations stayed above both the SARS-CoV-2 replicon assay EC_90_ of 36.1 ng/mL (70 nM) and the plasma binding-adjusted (PBA) SARS-CoV-2 replicon assay EC_90_ (PBA EC_90_) of 95 ng/mL for the 11 h time interval at 250 mg/kg, 500 mg/kg, and 1000 mg/kg (Figure 2), suggesting that twice-a-day (BID) dosing at a minimum dose of 250 mg/kg would approximately maintain the plasma levels in the therapeutic window for a full 24 h period. Additionally, therapeutic drug monitoring at the termination of the mouse efficacy studies demonstrated plasma levels above the PBA EC_90_ at troughs for the 500 mg/kg- and 1000 mg/kg-treated mice (Figure S7a). The PBA EC_90_ was calculated by dividing the SARS-CoV-2 replicon assay EC_90_ by the unbound mouse plasma fraction (fup) of 0.379. The measurement of plasma and lung concentrations at the 0.5 h time point after an oral dose of 500 mg/kg showed a lung-to-plasma ratio of 1:2, indicating comparable levels of MK-7845 in both the plasma and lung tissues (Figure S7b).

### 3.3. MK-7845 Reduced the SARS-CoV-2 Infection in a K18-hACE2 Transgenic Mouse Model

**Figure 3:**
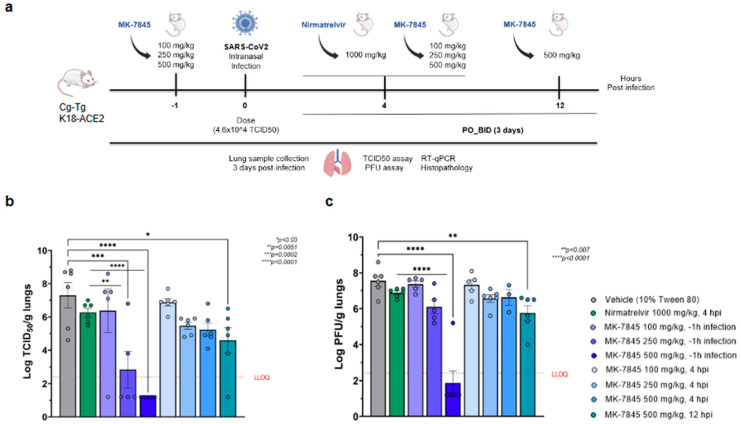
In vivo efficacy of MK-7845 against SARS-CoV-2 in K18-hACE2 mouse model of infection. (**a**) Schematic representation of mouse treatment and challenge. Transgenic K18-hACE2 mice that were 8–10 weeks old were challenged intranasally with SARS-CoV-2 USA-WA1/2020 (infection dose 4.6 × 10^4^ TCID_50_/mouse, *n* = 5–6 per group). MK-7845 or the vehicle (10% Tween 80) were administered orally twice a day for 3 days at doses of 100 mg/kg, 250 mg/kg, and 500 mg/kg 1 h prior to infection and 4 hpi, and 500 mg/kg 12 hpi. Nirmatrelvir was administered at 1000 mg/kg 4 hpi. Mice were euthanized 3 dpi and lung samples were collected for TCID_50_, plaque forming unit (PFU) assay, RT-qPCR, and histopathological analysis. Viral lung burdens at 3 dpi are represented as log 10 mean of TCID_50_ (**b**) and PFU (**c**) per gram of lung tissue ± SEM. Samples with values below the LLOQ were assigned a value half of LLOQ for analyses. The outliers below the LLOQ from the MK-7845 100 mg/kg 1 h prior to infection and 500 mg/kg 12 hpi groups were excluded from the analysis. Statistical significance was evaluated by one-way ordinary ANOVA with Dunnett’s multiple comparisons test.

The K18-hACE2 transgenic mouse model is genetically engineered to express the human angiotensin-converting enzyme 2 (ACE2) receptor under the control of the cytokeratin-18 promoter, which enables susceptibility to SARS-CoV-2 infection [24]. We recently published preliminary data in a study that evaluated the dose-response of MK-7845 in this model. The administration of MK-7845 in the study was performed orally, beginning at 1 h prior to infection and 9 hpi on day 0, with subsequent administrations twice a day for 3 days with doses of 100 mg/kg, 250 mg/kg, and 500 mg/kg [12]. We further evaluated the therapeutic effect of MK-7845 by initiating the administration of the compound at different post-infection time-points (4 hpi and 12 hpi) on day 0 in the current study. A schematic design of the study is represented in Figure 3a.

The body weight of the K18-hACE2 mice was monitored at baseline and following intranasal infection with 4.6 × 10^4^ of the TCID_50_ SARS-CoV-2 USA-WA1/2020. At 3 dpi, there were slight decreases in body weight among the vehicle-control group and most of the treated groups (Figure S8a). Unexpectedly, mice treated with 500 mg/kg of MK-7845 (12 hpi dosing group) gained weight from day 1 through day 3 (Figure S8a). A biological explanation associated with treatment for this result is unknown, as both the 4 hpi and 12 hpi groups had similar mean lung burdens following therapy (Figure 3). Regardless, this unanticipated result has little impact on the efficacy evaluation of MK-7845 in this in vivo test system.

The evaluation of viral lung burdens, measured by TCID_50_ and PFU assay, revealed a high level of virus in the vehicle-treated group (7–8 logs), and in the positive-control group treated with nirmatrelvir at 1000 mg/kg BID (~6 logs) (Figure 3b,c). However, dosing MK-7845 at 250 mg/kg and 500 mg/kg 1 h before infection significantly reduced viral loads by ~5 and 6 logs (*p* ≤ 0.002 and *p* < 0.0001, respectively), compared to the vehicle control group. Notably, MK-7845 also demonstrated significant 3.4 log and 5 log reductions in the viral burdens versus the nirmatrelvir group at this time point (*p* ≤ 0.005 and *p* < 0.0002, respectively). There was no significant reduction in viral load observed in the pulmonary compartment when MK-7845 dose administration was initiated at 4 hpi and 12 hpi (Figure 3b). The efficacy of the treatment with MK-7845 at 500 mg/kg 1 h before infection was further confirmed by a lung burden reduction of ~5–6 log PFU/g lungs (*p* < 0.0001) measured by PFU assay, compared to both the vehicle-control and nirmatrelvir-treated groups (Figure 3c). Like the TCID_50_ results, MK-7845 administered at 4 hpi and 12 hpi did not show a significant reduction in viral load compared to the vehicle-control group determined by PFU assay (Figure 3c). These results are in concordance with the levels of viral genomic RNA obtained from lung tissue (Figure S9a).

A semi-quantitative histopathological analysis was conducted on H&E-stained lung tissue from all mice (Figure S10). The histopathological evaluation showed that, in the vehicle group, mice developed mild to moderate pulmonary pathology caused by the viral replication in the lungs upon SARS-CoV-2 infection (Figure S10a–d). The lung pathology was similar in the nirmatrelvir- (1000 mg/kg), Figure S11a,b, and MK-7845- (100 mg/kg) treated groups when dosed 1 h prior to infection or 4 hpi and 12 hpi, while it was significantly reduced in 250 mg/kg- and 500 mg/kg-treated mice when dosed at 4 hpi (Figure S10e,f). Additional information from the histopathological analysis is provided in Figure S12a and Table S3.

### 3.4. MK-7845 Reduced the MERS-CoV Infection in a K18-hDPP4 Transgenic Mouse Model

**Figure 4:**
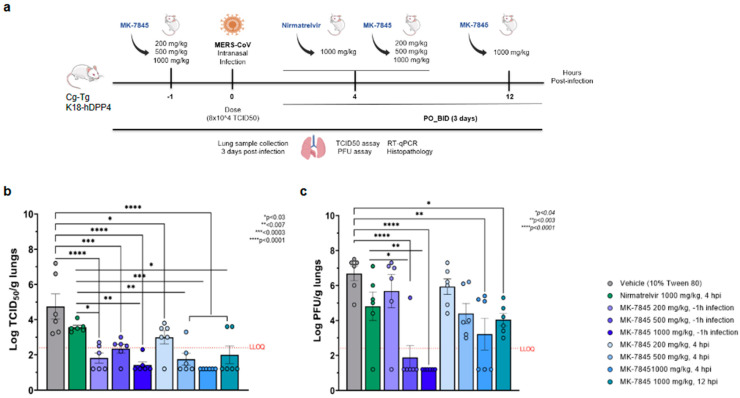
In vivo efficacy of MK-7845 against MERS-CoV in K18-hDPP4 mouse model of infection. (**a**) Schematic representation of mouse treatment and challenge. Transgenic K18-hDPP4 mice (7–9 weeks) were challenged intranasally with MERS-CoV EMC 2012 (infection dose 8.0 × 10^4^ TCID_50_/mouse, *n* = 6 per group). MK-7845 or the vehicle (10% Tween 80) were administered orally BID for 3 days at doses 200 mg/kg, 500 mg/kg, and 1000 mg/kg 1 h prior to infection and 4 hpi, and at 1000 mg/kg 12 hpi, while nirmatrelvir was administered at 1000 mg/kg 4 hpi. Mice were euthanized at 3 dpi and lung samples were collected for TCID_50_, PFU assay, RT-qPCR, and histopathological analysis. Viral lung burdens at 3 dpi are represented as log10 means of TCID_50_ (**b**) and PFU assay (**c**) per gram of lung tissue ± SEM. Samples with values below the LLOQ were assigned a value half of LLOQ for analyses. The outliers below the LLOQ from the nirmatrelvir 1000 mg/kg 4 hpi and MK-7845 200 mg/kg 1 h prior to infection groups were excluded from the analysis. Statistical significance was evaluated by one-way ordinary ANOVA with Dunnett’s multiple comparisons test.

To assess the in vivo efficacy of MK-7845 against other coronaviruses, a MERS-CoV infection model was induced by the intranasal inoculation of 8.0 × 10^4^ TCID_50_ MERS-CoV EMC 2012 in K18-hDPP4 transgenic mice expressing the hDPP4 receptor [17]. This model has been validated previously and exhibited significant weight loss, hypothermia, clinical manifestations as observed in humans, and death after 6–7 days of infection. In our study, MK-7845 was orally administered at doses of 200 mg/kg, 500 mg/kg, and 1000 mg/kg at 1 h prior to infection or 4 hpi, or at a dose of 1000 mg/kg at 12 hpi in K18-hDPP4 mice (Figure 4a). Vehicle-treated mice exhibited approximately 7% body weight loss at 3 dpi (Figure S8b). Overall, the compound-treated mice had a smaller decrease in body weight.

The mice treated with MK-7845 exhibited a significant dose-response reduction in lung viral burdens in both the prophylactic and therapeutic treatment groups (Figure 4b,c). All the prophylactic regimens of MK-7845 demonstrated protective effects against MERS-CoV infection compared to the vehicle-treated control mice. When administered orally at 1 h prior to infection, doses of 200 mg/kg, 500 mg/kg, and 1000 mg/kg MK-7845 resulted in a significant reduction in viral load compared to the vehicle group, with differences of ~3 log (*p* < 0.0001), ~2.4 log (*p* ≤ 0.0002), and ~3.3 log (*p* < 0.0001), respectively (Figure 4b). The effectiveness of MK-7845 doses administered 4 hpi was also demonstrated with reductions of ~2 log (*p* ≤ 0.01), ~3 log (*p* < 0.0001), and ~4 log (*p* < 0.0001), respectively, as measured by TCID_50_. MK-7845 demonstrated efficacy even when administered 12 hpi at a dose of 1000 mg/kg. In comparison to the vehicle-control group, MK-7845 at this dose resulted in a 3 log TCID_50_ (*p* ≤ 0.0001) reduction in the viral burden (Figure 4b). Furthermore, when compared to the nirmatrelvir group, MK-7845 achieved a 2-log reduction (*p* ≤ 0.01) and a 2.2 log reduction (*p* ≤ 0.001) in viral burden when administered at doses of 200 mg/kg and 1000 mg/kg, respectively, at 1 h prior to infection. Additionally, at 4 hpi, MK-7845 doses of 500 mg/kg and 1000 mg/kg demonstrated significant reductions of ~2 log (*p* ≤ 0.007) and 2.4 log (*p* ≤ 0.0003), respectively, compared to the nirmatrelvir group. Even at 12 hpi, treatment with MK-7845 at a 1000 mg/kg dose resulted in a significant ~2 log (*p* ≤ 0.03) reduction in viral burden (Figure 4b).

The efficacy of the treatment was further confirmed by PFU assay. Treatment with 500 mg/kg of MK-7845 prior to infection resulted in a substantial decrease in viral burden by ~6 log (*p* < 0.0001), compared to the vehicle-treated mice (Figure 4c). Moreover, the highest dose of MK-7845 (1000 mg/kg) at 1 h prior to infection allowed a recovery from infection as no plaques were formed after 72 h of incubation (*p* < 0.0001), as seen shown in Figure 4c. A significant clearance of infection was also observed in mice treated with MK-7845 at 1000 mg/kg when administered 4 hpi (*p* < 0.003) and 12 hpi (*p* < 0.04) (Figure 4c). In comparison to nirmatrelvir, MK-7845 at 500 mg/kg and 1000 m/kg showed 2.9 (*p* < 0.003) and 3.6 (*p* < 0.0002) log reductions in viral load, respectively. The viral genomic RNA was also evaluated in lung tissue by RT-PCR, but we did not observe a significant reduction (Figure S9b).

**Figure 5:**
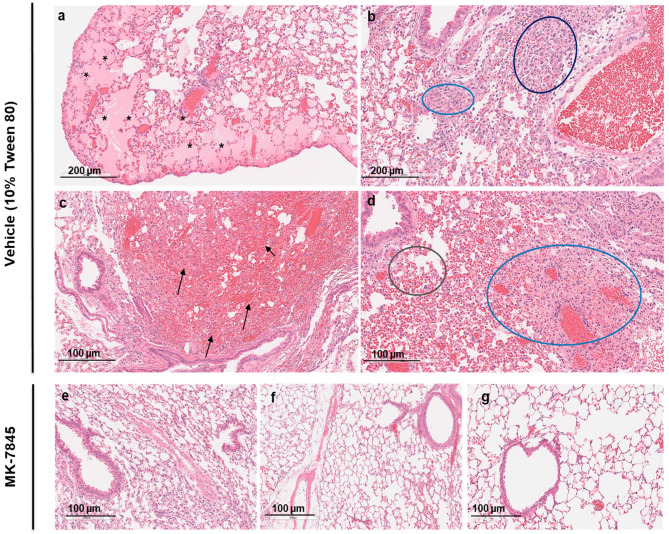
Histopathological analysis of MERS-CoV infection in K18-hDPP4 mice treated with MK-7845. Representative images showing lung histopathological evaluation in MERS-CoV-infected mice in vehicle control (**a**–**d**) and 500 mg/kg (**e**) and 1000 mg/kg (**f**,**g**) MK-7845-treated mice groups. H&E-stained lung sections showing MERS-CoV infection represented by multifocal to patchy consolidation in the lung, with perivascular and peribronchial inflammation and alveolar edema in vehicle control. Tissue sections show hemorrhage (arrows), alveolar edema (asterisk), perivascular infiltration (dark blue circle), inflammation (light blue and gray circle), and cell debris.

The histopathology induced by MERS-CoV infection was assessed in H&E-stained lung sections using a semi-quantitative histopathological analysis. The damage was characterized by various features including perivascular edema, hemorrhage, thrombosis, cell debris in lymphatics, and cellular inflammation (Figure 5). The vehicle-group showed multifocal hemorrhage, pulmonary edema in the alveoli, and cellular inflammation in 34–66% of the lung field, localized to multifocal cellular debris in lymphatics, with vascular thrombi formation within the areas of consolidation (Figure 5a–d). A similar pathology for alveolar and cellular inflammation was observed in the nirmatrelvir-treated group but with less vascular thrombi formation (Figure S11c,d). In all groups of mice treated with MK-7845, there was a notable reduction in lung pathology characterized by normal to localized alveolar edema, inflammation, and reduced cell debris in lymphatics with occasional vascular thrombi formation, particularly in mice that received MK-7845 at doses of 500 mg/kg and 1000 mg/kg at both 1 h prior and 4 hpi (Figure 5e,f).

## 4. Discussion

The SARS-CoV-2 virus was first discovered in China in 2019 [25] and, as of November 2023, there have been close to 800 million confirmed cases of COVID-19 with around 7 million deaths reported to the WHO (https://covid19.who.int; accessed on 10 January 2024). Remdesivir, known as Veklury^®^, a prodrug of an adenosine analogue that inhibits viral replication via incorporation by the RNA-dependent RNA polymerase (RdRp) enzyme previously studied for activity against HCV and filoviruses, emerged as a direct-acting antiviral treatment against SARS-CoV-2 in 2020. Remdesivir must be administered intravenously, making timely treatment in an outpatient setting more challenging than orally administered drugs.

As the pandemic continued, orally administered direct-acting antivirals emerged as treatment options for patients suffering with SARS-CoV-2 infections, including Lagevrio^®^ (molnupiravir) and Paxlovid^®^ (ritonavir-boosted nirmatrelvir). Both are indicated for mild to moderate COVID-19 in adults who do not require supplemental oxygen and who are at increased risk for progressing to severe COVID-19. Molnupiravir is an oral prodrug of beta-D-N4-hydroxycytidine (NHC), a ribonucleoside that functions by uptake into replicating viral RNA by viral RNA-dependent RNA-polymerases, thereby causing viral mutations and viral error catastrophe [26,27]. Nirmatrelvir, a covalent reversible inhibitor of SARS-CoV2 3CLPro, has been shown to contribute to a significant reduction in hospitalization rates and deaths by 80–90% among patients with COVID-19. However, nirmatrelvir requires pharmacokinetic boosting with the cytochrome P450 3A4 inhibitor ritonavir, which can result in significant drug–drug interactions with more than thirty drugs, many taken by those with risk factors for severe COVID-19.

More recently, ensitrelvir (S-217622), a non-covalent SARS-CoV-2 3CLPro inhibitor, received emergency regulatory approval in Japan for patients not at high risk for severe illness. Ensitrelvir is currently under evaluation in a Phase III clinical trial in the United States [28]. Direct-acting antivirals complement the available vaccines and therapeutic antibodies for SARS-CoV-2, which target the spike protein; however, the high level of mutations observed in the multiple subvariants that have arisen during the pandemic have affected their efficacy [29]. Even when vaccination is still recommended as the best strategic response, safe and effective new treatments are needed for patients infected with SARS-CoV-2.

Another coronavirus, MERS-CoV, was first identified in Saudi Arabia in 2012. While SARS-CoV-2 has proven to be more contagious, MERS-CoV has a higher case fatality rate as it has one-third mortality among those infected (Middle East respiratory syndrome coronavirus (MERS-CoV) (who.int)). According to the CDC, currently, there are no approved treatments or vaccines for MERS-CoV (MERS Prevention & Treatment | CDC) and the primary approach for managing MERS-CoV infection is to focus on symptom relief. With the high potential for the emergence of novel coronaviruses of concern, there is a need to develop new broad-spectrum antivirals to treat coronavirus-induced infections. Ideally, these new antivirals for pandemic preparedness will couple ease of administration, improved efficacy, and better pharmacological properties with broad-spectrum anti-coronavirus activity and a lower risk of drug–drug interactions.

Amongst the potential drug targets for broad-spectrum coronavirus antivirals, the main protease (3CLPro, Mpro, nsp5) is a virally encoded cysteine protease considered a key drug target due to its fundamental role in the cleavage and maturation of proteins involved in viral replication [30,31]. Amino acid sequence alignments indicate that the similarity of the 3CLPro of SARS-CoV, SARS-CoV-2, and MERS-CoV can be as high as 96.1% [32], with a high degree of structural similarity conserved across the coronavirus family [33]. These observations suggest that 3CLPro inhibitors could be explored as broad-spectrum coronavirus antivirals. The importance of this target is highlighted by the high level of interest within the scientific community [34,35,36]. Previously, the peptidomimetic inhibitors of enterovirus 3C protease have been reported as inhibitors of 3CLPro of SARS-CoV and MERS-CoV [37], with IC_50_ values from 0.2 nM to 700 nM and from 1700 nM to 4700 nM, respectively. More recently, another group developed several specific non-covalent inhibitors of 3CLPro and reported WU-04 as a broadly active compound with potency against wild-type SARS-CoV-2 and Omicron subvariants and MERS-CoV, as well as similar in vivo activity compared to nirmatrelvir in K18-hACE2 mice after oral administration [38].

One common strategy for the design of 3CLPro inhibitors involves mimicking the peptide substrates of the proteases [39]. The protein lumen of the SARS-CoV-2 3CLPro substrate-binding channel contains six subsites. Among them, S1’ is an important site for appending a covalent warhead that can interact with the Cys145-His41 catalytic dyad, wherein Cys145 functions as a nucleophile and His41 serves as the proton donor/acceptor [40]. Boceprevir attracted our attention due to its good pharmacokinetic and pharmacodynamic properties in humans and sufficient bioavailability to enable oral dosing, although it only exhibits modest activity versus SARS-CoV-2 [41]. Starting from a hit identified during a targeted screen, medicinal chemistry efforts led to MK-7845, which introduces a novel substitution pattern at the P1 position, providing improved activity against SARS-CoV-2 3CLPro [12]. An X-ray co-crystal structure of MK-7845 bound to SARS-CoV-2 3CLPro (PDB 8UTE) showed a nucleophilic attack of Cys145 on the reactive ketoamide, forming a covalent adduct, with the subsequent hydroxyl group of the tetrahedral intermediate within H-bonding distance of the catalytic His41. Jump dilution studies have aided in classifying MK-7845 as a reversible covalent inhibitor of 3CLPro.

In this study, we investigated MK-7845 for its in vitro potency using enzymatic, replicon, and cellular viral infectivity assays. A P-GP inhibitor was used at 2000 nM/well in all the in vitro assays performed in Vero E6+TMPRSS2 cells, due to the relatively high expression of P-GP in this cell line. The addition of elacridar to Vero E6+TMPRSS2 cells was not toxic (Figure S13a) and did not show an effect on the infection when using SARS-CoV-2 or MERS-CoV virus (Figure S13b,c), but remdesivir and nirmatrelvir showed a significant shift in activity in the presence versus the absence of the P-GP inhibitor (Figure S13d,e). MK-7845 showed potent in vitro activity against representative members of the human coronavirus family (hCoV-229E, hCoV-OC43, several SARS-CoV-2 Omicron subvariants, and MERS-CoV), as shown in Table 1 and Table 2. During this study, JN.1 was reported as the most prevalent Omicron subvariant, according to the CDC (CDC COVID Data Tracker), among SARS-CoV-2-related infections, with a 95% prevalence. All the SARS-CoV-2 Omicron strains carry P132H, one of the most prevalent 3CLPro missense mutations in the nsp5. However, this mutation does not confer antiviral resistance, as MK-7845 previously showed similar biochemical potency against the P132H mutant enzyme [12] and exhibited a potent inhibition of infection of Omicron subvariants in in vitro assays. MK-7845 was additionally investigated against 3CLPro from hCoV-NL63, HKU1, and MERS-CoV, which indicates the potential for molecules like MK-7845 to have broad-spectrum activity against coronaviruses.

To investigate whether the promising in vitro activity of MK-7845 translates to in vivo efficacy, we first conducted pharmacokinetic studies in healthy CD-1 mice to determine the dose selection for the efficacy study. Oral dose escalation pharmacokinetic studies in CD-1 mice and therapeutic drug monitoring in infected mice demonstrated a significant oral exposure of MK-7845 and plasma concentrations above the PBA EC90 for the entire 24 h period using BID doses between 100 mg/kg and 1000 mg/kg. Additionally, dose projections for MK-7845 determined in previously published studies estimated a reasonable clinical dose of 220 mg/kg BID, based upon preclinical PK studies in rats, dogs, and monkeys, without the need for co-administration of a CYP-450 inhibitor [12]. Our results from efficacy studies in transgenic mouse models infected with either SARS-CoV-2 or MERS-CoV demonstrated that MK-7845 significantly reduces viral loads in the lungs in both coronavirus models at 3 dpi, particularly when using the highest dose. This reduction in viral burden is accompanied by an improvement in associated lung histopathology changes. RT-PCR and histological examinations were performed at 3 days post-infection. While RT-PCR detects viral RNA, which can persist even after the virus is no longer infectious [21], histology examines tissues for pathology secondary to the constellation of infection, viral replication, and the host immune response, which results in observable cellular and tissue damage, as well as immune cell infiltration [24]. These processes may not be temporally aligned with the peak viral burden, potentially explaining the difference between the detectable RT-PCR and histological finding.

Other studies of these models have shown hallmarks of disease such as substantial weight loss and more severe lung histopathological changes, including additional immune cell infiltration, typically manifesting 4–7 dpi [17,24,42]. Thus, we recognize the need for additional experiments to evaluate both the prophylactic and therapeutic efficacy of MK-7845 in the context of the progressive inflammatory process that continues to occur following 3 dpi in these respiratory infection models, which can culminate in a severe viral pneumonia.

## 5. Conclusions

MK-7845 demonstrated potent in vitro activities against various pandemic and endemic hCoV. Moreover, it exhibited significant in vivo efficacy in rodent models of SARS-CoV-2 and MERS-CoV infection. These findings, coupled with physicochemical properties and a preclinical pharmacokinetic profile that predicts a human oral dose without the need for boosting by CYP3A4 inhibition, suggest that MK-7845 or similar compounds could be progressed as broad-spectrum antivirals for SARS-CoV-2 and pandemic readiness.

## Figures and Tables

**Table 1:** Biochemical and cellular activity of MK-7845. (a) Biochemical activity using FRET-based enzymatic assays with recombinant coronavirus 3CLPro enzymes; (b) EC_50_ values determined by cell-based CPE assays for endemic coronaviruses; (c) in vitro EC_50_ antiviral activity of MK-7845 against SARS-CoV-2 and MERS-CoV, using replicon-based cellular assays and whole-cell based assay with live viruses. ^1^ SARS-CoV-2 replicon construct pSMART BAC-T7-scv2-replicon; ^2^ SARS-CoV-2 Lineage B.1.1.529, Omicron variant; ^3^ MERS-CoV EMC 2012; ^4^ MERS-CoV icMERS-CoV-RFP-ΔORF5.

**a. Biochemical-enzymatic based assay**					
**Viral protease**	**Coronavirus**	**MK-7845** **IC50 Geomean (nM)**		**Nirmatrelvir** **IC50 Geomean (nM)**	
SARS-CoV-1 3CLPro	Beta	19.2		20.3	
SARS-CoV-2 3CLPro	Beta	8.7		10.3	
MERS-CoV 3CLPro	Beta	318		118	
hCoV-229E 3CLPro	Alpha	307		57	
hCoV-OC43 3CLPro	Beta	17		6.2	
hCoV-HKU1 3CLPro	Beta	25.5		9.7	
hCoV-NL63 3CLPro	Alpha	167		124	
**b. Cell-based CPE assay**					
**Virus**	**Coronavirus**	**MK-7845** **EC50 Geomean (nM)**		**Nirmatrelvir** **EC50 Geomean (nM)**	
hCoV-229E	Alpha	3150		473	
hCoV-OC43	Beta	370		163	
None	-	>36,000		>36,000	
**c. Replicon-based and whole-cell based assays**					
**Coronavirus**	**Strain**	**EC50 Geomean (nM)**			
		**MK-7845**	**Nirmatrelvir**		**Remdesivir**
SARS-CoV-2	Wuhan ^1^ (Replicon)	15	10		270
	B.1.1.529 ^2^ (Live virus)	140	70		170
MERS-CoV	EMC 2012 ^3^ (Replicon)	270	60		780
	icMERS-CoV-RFP-ΔORF5 ^4^ (Live virus)	390	70		150

**Table 2:** In vitro profile of MK-7845 activity against coronaviruses using whole-cell-based assays. Vero E6+TMPRSS2 cells were pretreated with MK-7845 in comparison with nirmatrelvir and remdesivir, used as reference drugs. The P-GP inhibitor was dispensed into the cells at 2000 nM/well. After 2 h incubation, cells were infected with each corresponding virus at MOI 0.5 (a) or MOI 0.1 (b). The data shown is the mean and SD from three independent experiments.

		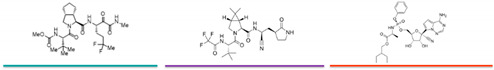											
**Virus**	**Strain**	**MK-7845**				**Nirmatrelvir**				**Remdesivir**			
		**Mean ± SD (nM)**				**Mean ± SD (nM)**				**Mean ± SD (nM)**			
		**IC50**	**EC50**	**EC90**	**SI**	**IC50**	**EC50**	**EC90**	**SI**	**IC50**	**EC50**	**EC90**	**SI**
**SARS-CoV-2** **Omicron**	B.1.1.529 ^a^	120 ± 6	138 ± 8	185 ± 18	>725	73 ± 13	66 ± 16	90 ± 36	>1527	169 ± 30	155 ± 11	166 ± 12	129
	BA.2 ^a^	212 ± 53	182 ± 57	260 ± 90	>549	78 ± 6	105 ± 69	123 ± 78	>957	93 ± 7	90 ± 47	212 ± 36	223
	BA.5 ^a^	168 ± 14	235 ± 93	408 ± 23	>426	96 ± 11	152 ± 6	199 ± 39	>660	133 ± 36	141 ± 15	200 ± 33	142
	BQ.1 ^a^	81 ± 23	191 ± 6	328 ± 10	>524	122 ± 6	50 ± 10	110 ± 67	>2020	144 ± 5	150 ± 8	178 ± 11	133
	BF.5 ^a^	229 ± 7	229 ± 7	518 ± 59	>304	133 ± 8	71 ± 20	98 ± 46	>1408	105 ± 13	154 ± 1	154 ± 1	130
	XBB.1.5 ^a^	275 ± 76	183 ± 62	372 ± 58	>546	42 ± 8	50 ± 23	75 ± 32	>2020	189 ± 73	149 ± 4	182 ± 7	135
	XBB.1.16.1 ^b^	158 ± 8	156 ± 13	234 ± 59	>641	89 ± 25	76 ± 27	107 ± 54	>1316	92 ± 6	110 ± 5	198 ± 2	183
	EG.5.1 ^b^	136 ± 6	176 ± 11	379 ± 14	>568	102 ± 64	104 ± 60	124 ± 80	>966	87 ± 11	163 ± 2	194 ± 2	123
	JN.1 ^b^	87 ± 36	98 ± 42	135 ± 28	>1020	91 ± 56	93 ± 54	109 ± 63	>1075	87 ± 30	94 ± 52	105 ± 54	214
**MERS-CoV**	EMC 2012 ^a^	362 ± 65	463 ± 8	541 ± 10	>216	117 ± 23	88 ± 30	164 ± 10	>1136	167 ± 33	110 ± 18	240 ± 93	182

## Data Availability

Data are contained within the article.

## Supplementary Materials

The following supporting information can be downloaded at https://www.mdpi.com/article/10.3390/v16071158/s1. Figure S1: Nirmatrelvir dose evaluation against SARS-CoV-2 and MERS-CoV; Figure S2: Characterization of MK-7845 interaction with SARS-CoV-2 3CLPro; Figure S3: Cytotoxic effect of MK-7845 in Vero E6+TMPRSS2 cells; Figure S4: IC_50_ dose-response curves of MK-7845 in vitro inhibitory activity against SARS-CoV-2; Figure S5: EC_50_ dose-response curves of MK-7845 in vitro inhibitory activity against SARS-CoV-2; Figure S6: IC_50_ & EC_50_ dose-response curves of MK-7845 in vitro inhibitory activity against MERS-CoV; Figure S7: MK-7845 trough levels in MERS-CoV model. (a) Trough plasma concentration of MK-7845 collected 12 h after the final dose in MERS-CoV infected mice; (b) Trough plasma and tissue concentrations of MK-7845 at 0.5 h after a single oral dose of 500 mg/kg in CD-1 mice; Figure S8: Body weight curves of K18-hACE2 mice infected with SARS-CoV-2 (a) and K18-hDPP4 mice infected with MERS-CoV (b); Figure S9: Lung burden analysis by RT-qPCR after intranasal infection with SARS-CoV-2 (a) and MERS-CoV (b); Figure S10: Histopathology induced by viral replication is assessed in SARS-CoV-2 infected lung of transgenic K18-hACE2 mice treated with MK-7845; Figure S11: Nirmatrelvir efficacy against SARS-CoV-2 and MERS-CoV infection; Figure S12: Lung histopathology composite score. Semi-quantitative analysis of histopathological changes in H&E- stained mice lungs after SARS-CoV-2 (a) and MERS-CoV (b) infection; Figure S13: P-GP inhibitor evaluation of activity and cytotoxicity in Vero E6+TMPRSS2 cells; Table S1 GISAID EPI_SET_240702sw Additional Information; Table S2: Sequence of primers and probe used for SARS-CoV-2 (a) and MERS-CoV (b); Table S3: Histopathological analysis of the lungs of mice infected with SARS-CoV-2; Table S4: Histopathological analysis of the lungs of mice infected with MERS-CoV.

## References

[1] WHO (2014). Global Alert and Response Middle East Respiratory Syndrome Coronavirus (MERS-CoV)—Saudi Arabia.

[2] Qamar M.T.U., Alqahtani S.M., Alamri M.A., Chen L.L. (2020). Structural basis of SARS-CoV-2 3CLPro and anti-COVID-19 drug discovery from medicinal plants. J. Pharm. Anal..

[3] Kandwal S., Fayne D. (2023). Genetic conservation across SARS-CoV-2 non-structural proteins—Insights into possible targets for treatment of future viral outbreaks. Virology.

[4] Rut W., Groborz K., Zhang L., Sun X., Zmudzinski M., Pawlik B., Wang X., Jochmans D., Neyts J., Młynarski W. (2021). SARS-CoV-2 Mpro inhibitors and activity-based probes for patient-sample imaging. Nat. Chem. Biol..

[5] Jin Z., Du X., Xu Y., Deng Y., Liu M., Zhao Y., Zhang B., Li X., Zhang L., Peng C. (2020). Structure of Mpro from SARS-CoV-2 and discovery of its inhibitors. Nature.

[6] Joyce R.P., Hu V.W., Wang J. (2022). The history, mechanism, and perspectives of nirmatrelvir (PF-07321332): An orally bioavailable main protease inhibitor used in combination with ritonavir to reduce COVID-19-related hospitalizations. Med. Chem. Res..

[7] Hsu A., Granneman G.R., Bertz R.J. (1998). Ritonavir. Ritonavir. Clinical pharmacokinetics and interactions with other anti-HIV agents. Clin. Pharmacokinet..

[8] Vandyck K., Abdelnabi R., Gupta K., Jochmans D., Jekle A., Deval J., Misner D., Bardiot D., Foo C.S., Liu C. (2021). ALG-097111, a potent and selective SARS-CoV-2 3-chymotrypsin-like cysteine protease inhibitor exhibits in vivo efficacy in a Syrian Hamster model. Biochem. Biophys. Res. Commun..

[9] Fu L., Ye F., Feng Y., Yu F., Wang Q., Wu Y., Zhao C., Sun H., Huang B., Niu P. (2020). Both Boceprevir and GC376 efficaciously inhibit SARS-CoV-2 by targeting its main protease. Nat. Commun..

[10] Oerlemans R., Ruiz-Moreno A.J., Cong Y., Kumar N.D., Velasco-Velazquez M.A., Neochoritis C.G., Smith J., Reggiori F., Groves M.R., Dömling A. (2020). Repurposing the HCV NS3-4A protease drug boceprevir as COVID-19 therapeutics. RSC Med. Chem..

[11] Lam J.T., Jacob S. (2012). Boceprevir: A recently approved protease inhibitor for hepatitis C virus infection. Am. J. Health Syst. Pharm..

[12] Shurtleff V.W., Layton M.E., Parish C.A., Perkins J.J., Schreier J.D., Wang Y., Adam G.C., Alvarez N., Bahmanjah S., Bahnck-Teets C.M. (2024). Invention of MK-7845, a SARS-CoV-2 3CL protease inhibitor employing a novel difluorinated glutamine mimic. J. Med. Chem..

[13] Biacchesi S., Skiadopoulos M.H., Yang L., Murphy B.R., Collins P.L., Buchholz U.J. (2005). Rapid human metapneumovirus microneutralization assay based on green fluorescent protein expression. J. Virol. Methods..

[14] Mendoza E.J., Manguiat K., Wood H., Drebot M. (2020). Two Detailed Plaque Assay Protocols for the Quantification of Infectious SARS-CoV-2. Curr. Protoc. Microbiol..

[15] Tomar S., Johnston M.L., John S.E.S., Osswald H.L., Nyalapatla P.R., Paul L.N., Ghosh A.K., Denison M.R., Mesecar A.D. (2015). Ligand-induced Dimerization of Middle East Respiratory Syndrome (MERS) Coronavirus nsp5 Protease (3CLPro): Implications for nsp5 Regulation and the Development of Antivirals. J. Biol Chem..

[16] Iversen P.W., Eastwood B.J., Sittampalam G.S., Cox K.L. (2006). A comparison of assay performance measures in screening assays: Signal window, Z’ factor, and assay variability ratio. J. Biomol. Screen..

[17] Li K., Wohlford-Lenane C., Perlman S., Zhao J., Jewell A.K., Reznikov L.R., Gibson-Corley K.N., Meyerholz D.K., McCray P.B. (2016). Middle East Respiratory Syndrome Coronavirus Causes Multiple Organ Damage and Lethal Disease in Mice Transgenic for Human Dipeptidyl Peptidase 4. J. Infect. Dis..

[18] Moreau G.B., Burgess S.L., Sturek J.M., Donlan A.N., Petri W.A., Mann B.J. (2020). Evaluation of K18-hACE2 mice as a model of SARS-CoV-2 infection. Am. J. Trop. Med. Hyg..

[19] Jeong J.H., Chokkakula S., Min S.C., Kim B.K., Choi W.-S., Oh S., Yun Y.S., Kang D.H., Lee O.-J., Kim E.-G. (2022). Combination therapy with nirmatrelvir and molnupiravir improves the survival of SARS-CoV-2 infected mice. Antiviral Res..

[20] Owen D.R., Allerton C.M.N., Anderson A.S., Aschenbrenner L., Avery M., Berritt S., Boras B., Cardin R.D., Carlo A., Coffman K.J. (2021). An oral SARS-CoV-2 Mpro inhibitor clinical candidate for the treatment of COVID-19. Science.

[21] Corman V.M., A Müller M., Costabel U., Timm J., Binger T., Meyer B., Kreher P., Lattwein E., Eschbach-Bludau M., Nitsche A. (2012). Assays for laboratory confirmation of novel human coronavirus (hCoV-EMC) infections. Eurosurveillance.

[22] Zhang H., Zhou P., Wei Y., Yue H., Wang Y., Hu M., Zhang S., Cao T., Yang C., Li M. (2020). Histopathologic Changes and SARS-CoV-2 Immunostaining in the Lung of a Patient with COVID-19. Ann. Intern. Med..

[23] De Clercq E. (2006). Potential antivirals and antiviral strategies against SARS coronavirus infections. Expert Rev. Anti Infect. Ther..

[24] Winkler E.S., Bailey A.L., Kafai N.M., Nair S., McCune B.T., Yu J., Fox J.M., Chen R.E., Earnest J.T., Keeler S.P. (2020). SARS-CoV-2 infection of human ACE2-transgenic mice causes severe lung inflammation and impaired function. Nat. Immunol..

[25] Hao Y., Wang Y., Wang M., Zhou L., Shi J., Cao J., Wang D. (2022). The origins of COVID-19 pandemic: A brief overview. Transbound Emerg Dis..

[26] Zhou S., Hill C.S., Sarkar S., Tse L.V., Woodburn B.M.D., Schinazi R.F., Sheahan T.P., Baric R.S., Heise M.T., Swanstrom R. (2021). β-d-N4-hydroxycytidine Inhibits SARS-CoV-2 Through Lethal Mutagenesis but Is Also Mutagenic to Mammalian Cells. J. Infect. Dis..

[27] Kabinger F., Stiller C., Schmitzová J., Dienemann C., Kokic G., Hillen H.S., Höbartner C., Cramer P. (2021). Mechanism of molnupiravir-induced SARS-CoV-2 mutagenesis. Nat. Struct. Mol. Biol..

[28] Lin M., Zeng X., Duan Y., Yang Z., Ma Y., Yang H., Yang X., Liu X. (2023). Molecular mechanism of ensitrelvir inhibiting SARS-CoV-2 main protease and its variants. Commun. Biol..

[29] Prévost J., Finzi A. (2021). The great escape? SARS-CoV-2 variants evading neutralizing responses. Cell Host Microbe.

[30] Thiel V., Herold J., Schelle B., Siddell S.G. (2001). Viral Replicase Gene Products Suffice for Coronavirus Discontinuous Transcription. J. Virol..

[31] Ullrich S., Nitsche C. (2020). The SARS-CoV-2 main protease as drug target. Bioorg. Med. Chem. Lett..

[32] Liu Y., Liang C., Xin L., Ren X., Tian L., Ju X., Li H., Wang Y., Zhao Q., Liu H. (2020). The development of Coronavirus 3C-Like protease (3CLpro) inhibitors from 2010 to 2020. Eur. J. Med. Chem..

[33] Wu F., Zhao S., Yu B., Chen Y.-M., Wang W., Song Z.-G., Hu Y., Tao Z.-W., Tian J.-H., Pei Y.-Y. (2020). A new coronavirus associated with human respiratory disease in China. Nature.

[34] Kuzikov M., Costanzi E., Reinshagen J., Esposito F., Vangeel L., Wolf M., Ellinger B., Claussen C., Geisslinger G., Corona A. (2021). Identification of Inhibitors of SARS-CoV-2 3CL-Pro Enzymatic Activity Using a Small Molecule in Vitro Repurposing Screen. ACS Pharmacol. Transl. Sci..

[35] Mody V., Ho J., Wills S., Mawri A., Lawson L., Ebert M.C.C.J.C., Fortin G.M., Rayalam S., Taval S. (2021). Identification of 3-chymotrypsin-like protease (3CLPro) inhibitors as potential anti-SARS-CoV-2 agents. Commun. Biol..

[36] Narayanan A., Narwal M., Majowicz S.A., Varricchio C., Toner S.A., Ballatore C., Brancale A., Murakami K.S., Jose J. (2022). Identification of SARS-CoV-2 inhibitors targeting Mpro and PLpro using in-cell-protease assay. Commun. Biol..

[37] Kumar V., Shin J.S., Shie J.-J., Ku K.B., Kim C., Go Y.Y., Huang K.-F., Kim M., Liang P.-H. (2017). Identification and evaluation of potent Middle East Respiratory syndrome coronavirus (MERS-CoV) 3CLPro inhibitors. Antivir. Res..

[38] Hou N., Shuai L., Zhang L., Xie X., Tang K., Zhu Y., Yu Y., Zhang W., Tan Q., Zhong G. (2023). Development of Highly Potent Noncovalent Inhibitors of SARS-CoV-2 3CLpro. ACS Cent. Sci..

[39] Xiong M., Su H., Zhao W., Xie H., Shao Q., Xu Y. (2021). What coronavirus 3C-like protease tells us: From structure, substrate selectivity, to inhibitor design. Med. Res. Rev..

[40] Stoddard S.V., Stoddard S.D., Oelkers B.K., Fitts K., Whalum K., Whalum K., Hemphill A.D., Manikonda J., Martinez L.M., Riley E.G. (2020). Optimization Rules for SARS-CoV-2 Mpro Antivirals: Ensemble Docking and Exploration of the Coronavirus Protease Active Site. Viruses.

[41] Venkatraman S., Bogen S.L., Arasappan A., Bennett F., Chen K., Jao E., Liu Y.-T., Lovey R., Hendrata S., Huang Y. (2006). Discovery of (1R,5S)-N-[3-amino-1-(cyclobutylmethyl)-2,3-dioxopropyl]-3-[2(S)-[[[(1,1-dimethylethyl) amino] carbonyl] amino]-3,3-dimethyl-1-oxobutyl]-6,6-dimethyl-3-azabicyclo [3.1.0] hexan-2(S)-carboxamide (SCH 503034), a selective, potent, orally bioavailable hepatitis C virus NS3 protease inhibitor: A potential therapeutic agent for the treatment of hepatitis C infection. J. Med. Chem..

[42] Yinda C.K., Port J.R., Bushmaker T., Offei Owusu I., Purushotham J.N., Avanzato V.A., Fischer R.J., Schulz J.E., Holbrook M.G., Hebner M.J. (2021). K18-hACE2 mice develop respiratory disease resembling severe COVID-19. PLoS Pathog..

