# Supplementary material for: Novel Pan-Coronavirus 3CL Protease Inhibitor MK-7845: Biological and Pharmacological Profiling
Source: Viruses. 2024 Jul 18;16(7):1158. doi: 10.3390/v16071158 (PMC11281459; doi:10.3390/v16071158)
Supplement: Supplementary file 1 [file viruses-16-01158-s001.zip › viruses-3086696-supplementary.pdf]

SUPPLEMENTARY INFORMATION FOR PUBLICATION

Novel Pan-Coronavirus 3CL Protease Inhibitor MK-7845: Biological and Pharmacological Profiling

Nadine Alvarez [1,*], Gregory C. Adam [2,*], John A. Howe [2], Vijeta Sharma [1], Matthew D. Zimmerman [1], Enriko Dolgov [1], Risha Rasheed [1], Fatima Nizar [1], Khushboo Sahay [1], Andrew M. Nelson [1], Steven Park [1], Xiaoyan Zhou [2], Christine Burlein [2], John F. Fay [2], Daniel V. Iwamoto [2], Carolyn M. Bahnck-Teets [2], Krista L. Getty [2], Shih Lin Goh [2], Imad Salhab [2], Keith Smith [2], Christopher W. Boyce [2], Tamara D. Cabalu [2], Nicholas Murgolo [2], Nicholas G. Fox [2], Todd W. Mayhood [2], Valerie W. Shurtleff [2], Mark E. Layton [2], Craig A. Parish [2], John A. McCauley [2], David B. Olsen [2] and David S. Perlin [1]

[1] Center for Discovery and Innovation, Hackensack Meridian Health, 111 Ideation Way, Nutley, NJ 07110, USA; vijeta.sharma@hmh-cdi.org (V.S.); matthew.zimmerman@hmh-cdi.org (M.D.Z.); enriko.dolgov@hmh-cdi.org (E.D.); risha.rasheed@hmh-cdi.org (R.R.); fatimanasreen100@gmail.com (F.N.); khushboo0503@gmail.com (K.S.); andrewm.nelson@hmh-cdi.org (A.M.N.); steven.park@hmh-cdi.org (S.P.); david.perlin@hmh-cdi.org (D.S.P.)

[2] Merck & Co., Inc., Rahway, NJ 07065, USA; john.howe@merck.com (J.A.H.); xiaoyan_zhou@merck.com (X.Z.); christine_burlein@merck.com (C.B.); john_fay@merck.com (J.F.F.); daniel.iwamoto@merck.com (D.V.I.); carolyn_bahnck-teets@merck.com (C.M.B.-T.); krista_getty@merck.com (K.L.G.); shih.lin.goh@merck.com (S.L.G.); isalhab@gmail.com (I.S.); keith.smith@merck.com (K.S.); christopher.boyce2@merck.com (C.W.B.); tamara_cabalu@merck.com (T.D.C.); nicholas.murgolo@merck.com (N.M.); nicholas.fox@merck.com (N.G.F.); todd.mayhood@merck.com (T.W.M.); valerie.shurtleff@merck.com (V.W.S.); mark_layton@merck.com (M.E.L.); craig_parish@merck.com (C.A.P.); john_mccauley@merck.com (J.A.M.); david_olsen@merck.com (D.B.O.)

* Correspondence: nadine.alvarez@hmh-cdi.org (N.A.); gregory_adam@merck.com (G.C.A.)

CONTENTS

Figure S1 Nirmatrelvir dose evaluation against SARS-CoV-2 and MERS-CoV.
Figure S2 Characterization of MK-7845 interaction with SARS-CoV-2 3CLPro.
Figure S3 Cytotoxic effect of MK-7845 in Vero E6+TMPRSS2 cells.
Figure S4 IC_{50} dose-response curves of MK-7845 in vitro inhibitory activity against SARS-CoV-2.
Figure S5 EC_{50} dose-response curves of MK-7845 in vitro inhibitory activity against SARS-CoV-2.
Figure S6 IC_{50} & EC_{50} dose-response curves of MK-7845 in vitro inhibitory activity against MERS-CoV
Figure S7 MK-7845 trough levels in MERS-CoV model. a) Trough plasma concentration of MK-7845 collected 12 h after the final dose in MERS-CoV infected mice; b) Trough plasma and tissue concentrations of MK-7845 at 0.5 h after a single oral dose of 500 mg/kg in CD-1 mice.
Figure S8 Body weight curves of K18-hACE2 mice infected with SARS-CoV-2 (**a**) and K18-hDPP4 mice infected with MERS-CoV (**b**).
Figure S9 Lung burden analysis by RT-qPCR after intranasal infection with SARS-CoV-2 (**a**) and MERS-CoV (**b**).
Figure S10 Histopathology induced by viral replication is assessed in SARS-CoV-2 infected lung of transgenic K18-hACE2 mice treated with MK-7845.
Figure S11 Nirmatrelvir efficacy against SARS-CoV-2 and MERS-CoV infection.
Figure S12 Lung histopathology composite score. Semi-quantitative analysis of histopathological changes in H&E- stained mice lungs after SARS-CoV-2 **(a)** and MERS-CoV (**b**) infection.
Figure S13 P-GP inhibitor evaluation of activity and cytotoxicity in Vero E6+TMPRSS2 cells.

Table S1 GISAID EPI_SET_240702sw Additional Information.

Table S2 Sequence of primers and probes used for SARS-CoV-2 **(a)** and MERS-CoV **(b).**
Table S3 Histopathological analysis of the lungs of mice infected with SARS-CoV-2.
Table S4 Histopathological analysis of the lungs of mice infected with MERS-CoV.

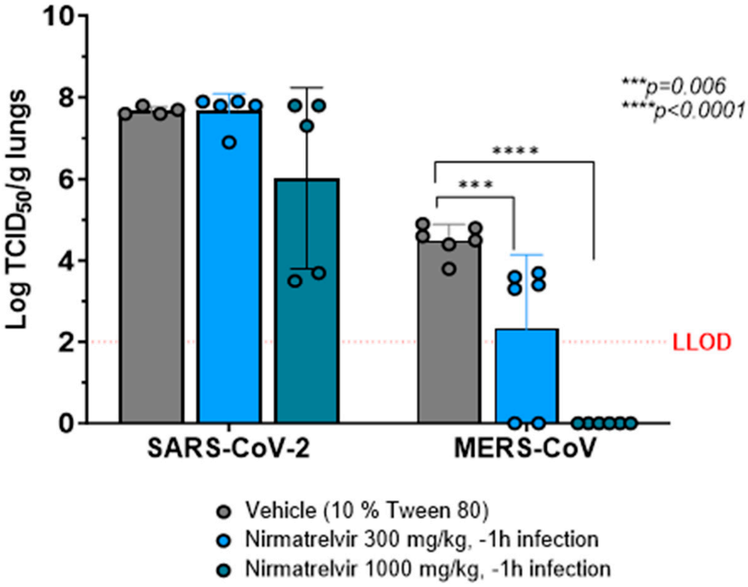

Figure S1 Prophylactic dose-response of nirmatrelvir against SARS-CoV-2 and MERS-CoV infection. Transgenic K18-hACE2 mice (n = 4-6) and K18-hDPP4 mice (n = 6) intranasally infected with SARS-CoV-2 WA/USA (BEI) and MERS-CoV (BEI) respectively, were treated with BID doses of vehicle (10% Tween 80) or nirmatrelvir (300 mg/kg or 1000 mg/kg), 1 h prior to infection. Graphs show lung viral titer as log10 of $TCID_{50}$ / gram ± SEM. Samples with values below the LLOQ were assigned a value half of LLOQ for analyses. Statistical significance was evaluated by one-way ordinary ANOVA with Dunnett's multiple comparisons test.

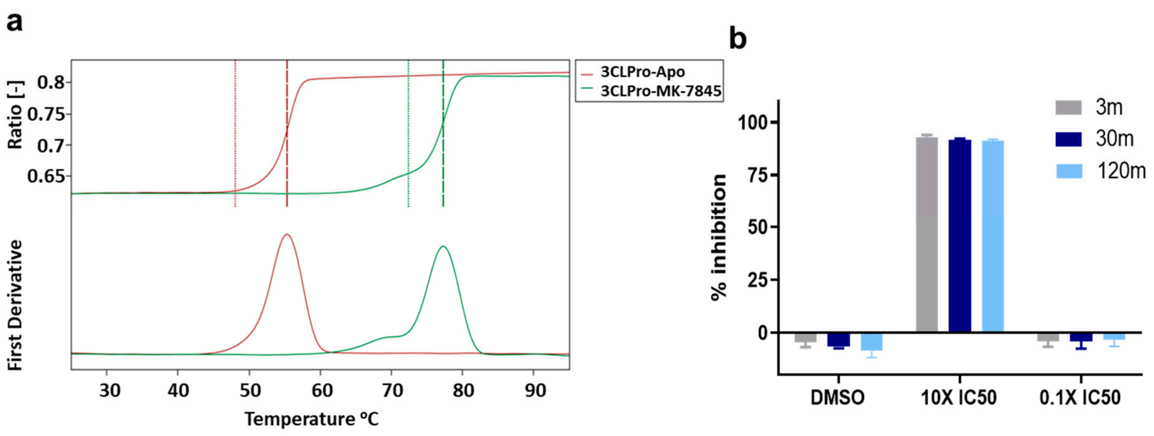

Figure S2 Characterization of interaction of MK-7845 with SARS-CoV-2 3CLPro. a) Comparison of the thermostability of 3CLPro measured by dynamic scanning fluorimetry of the apo form (*red lines*) and protein plus MK-7845 (*green line*) indicates stabilization by compound binding of 21.8°C. **b**) Jump dilution studies confirm a reversible interaction between MK-7845 and 3CLPro wherein enzyme was preincubated with 10x IC_{50} of MK-7845, then rapidly diluted to 0.1x IC_{50} and the percent inhibition measured at 3 min, 30 min and 120 min post-dilution.

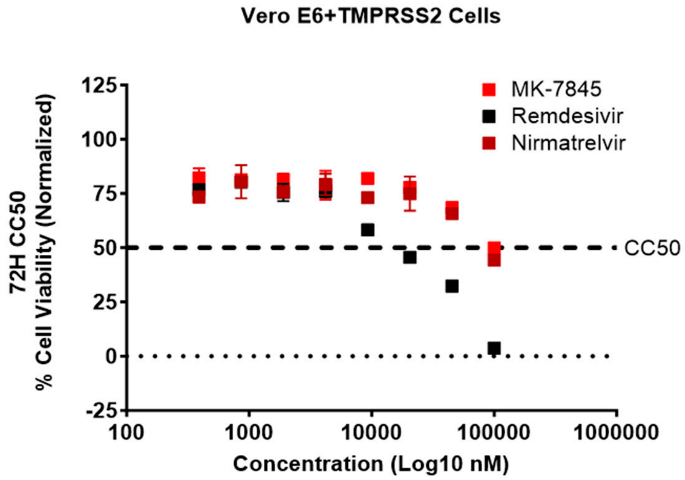

Figure S3 Cytotoxic effect of MK-7845 in Vero E6+TMPRSS2 cells. Vero E6+TMPRSS2 cells were treated with the test article (MK-7845) and the comparators (nirmatrelvir and remdesivir) during 72 h, in the presence of P-GP inhibitor (Elacridar) at 2,000 nM / well. All three compounds were evaluated using 8-points of titration and two-fold dilutions, with a range from 100,000 nM to 780 nM. The 50% cytotoxic concentration was determined in comparison with the untreated cells and expressed as CC_{50} values. Data shown are the mean and SD from three independent experiments.

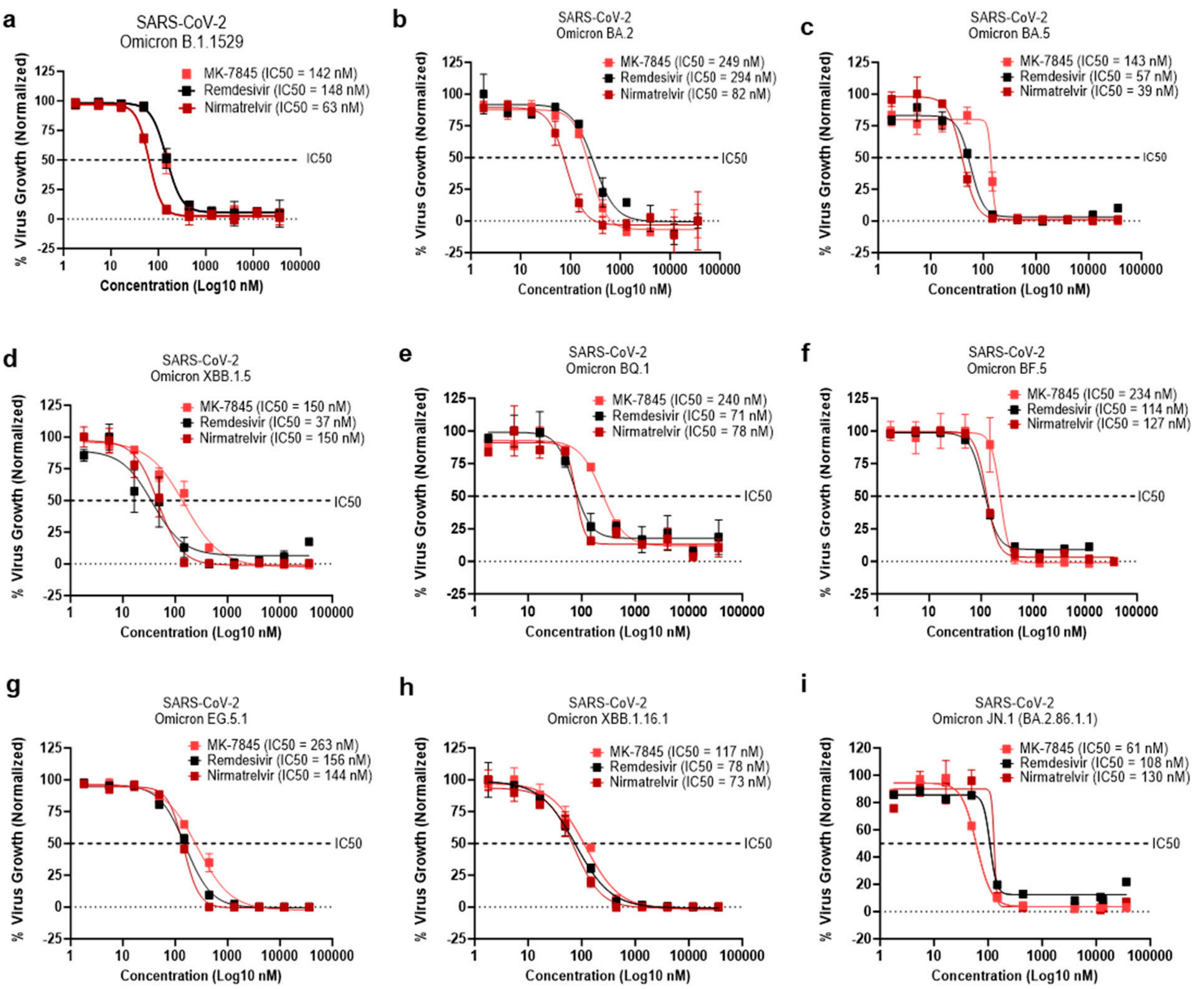

Figure S4 Representative IC_{50} dose-response curves of MK-7845 in vitro inhibitory activity against SARS-CoV-2 infection using different Omicron subvariants. Vero E6+TMPRSS2 cells were pretreated with compounds (10 doses using three-fold dilution from 36,000 nM to 1.8 nM) for 2 h at 37°C and 5% CO_2, followed by infection with MOI 0.5 and 48 h incubation. Data shown are the mean and SD from three independent experiments.

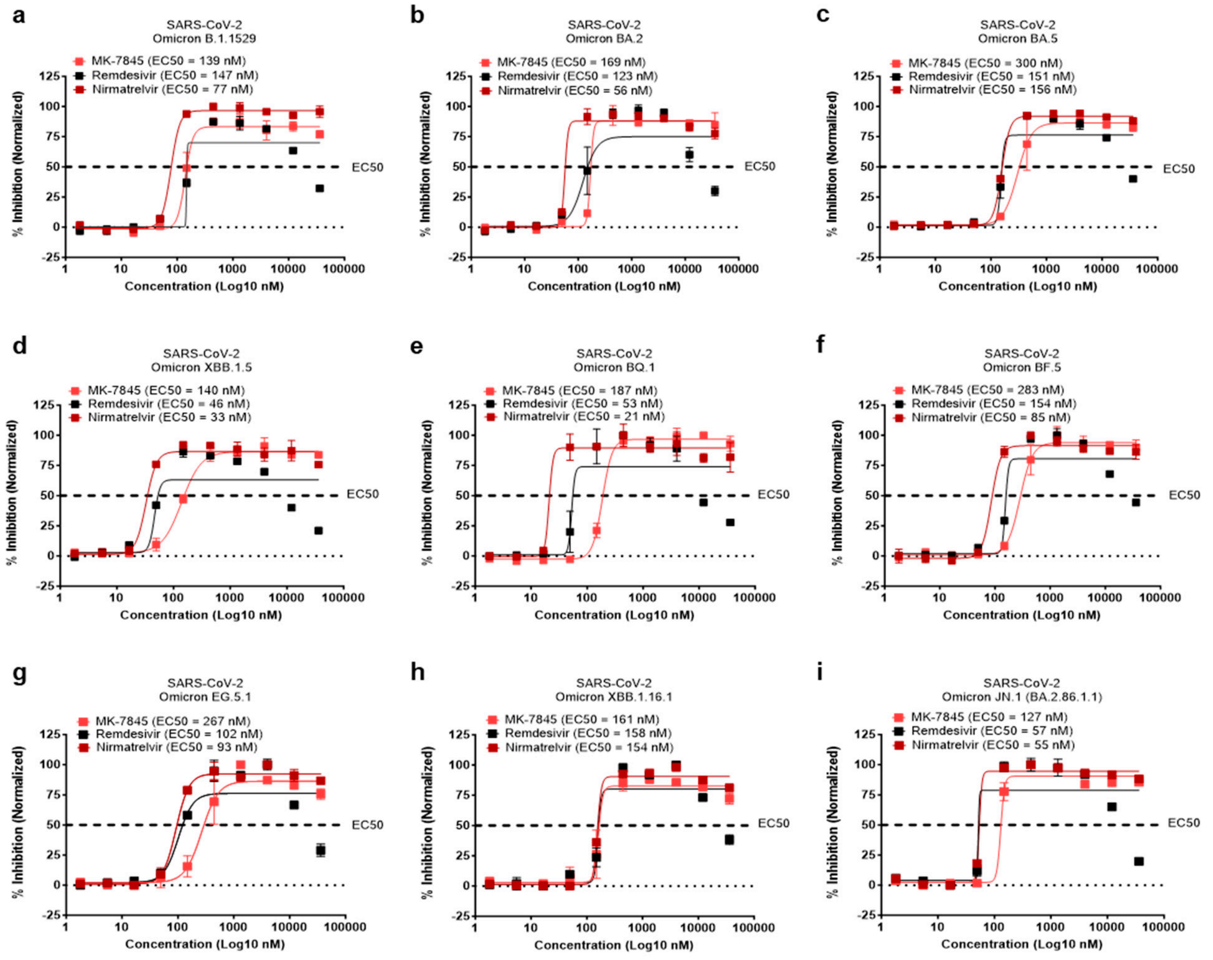

Figure S5 Representative EC_{50} dose-response curves of MK-7845 in vitro inhibitory activity against SARS-CoV-2 infection using different Omicron subvariants. Vero E6+TMPRSS2 cells were pretreated with compounds (10 doses using three-fold dilution from 36,000 nM to 1.8 nM) for 2 h at 37°C and 5% CO_2, followed by infection at MOI 0.5 and 72 h incubation. Data shown are the mean and SD from three independent experiments.

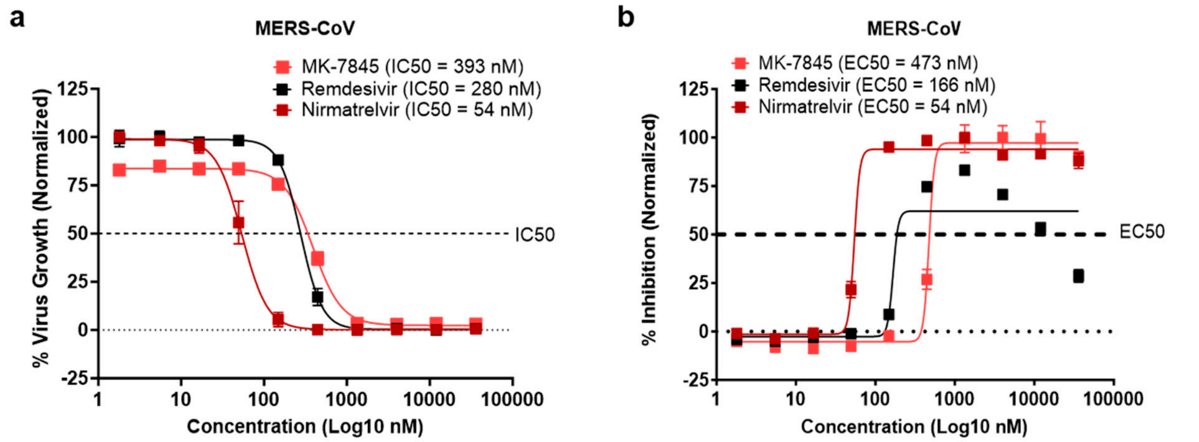

Figure S6 Representative IC_{50} and EC_{50} dose-response curves of MK-7845 in vitro inhibitory activity against MERS-CoV virus expressing RFP. Vero E6+TMPRSS2 cells were pretreated with compounds (10 doses using three-fold dilution from 36,000 nM to 1.8 nM) and Elacridar (P-GP inhibitor, 2,000 nM / well) for 2 h at 37°C and 5% CO_2, followed by infection at MOI 0.5. The inhibitory activity was evaluated at 48 h (**a**) and 72 h (**b**) after infection. Data shown are the mean and SD from three independent experiments.

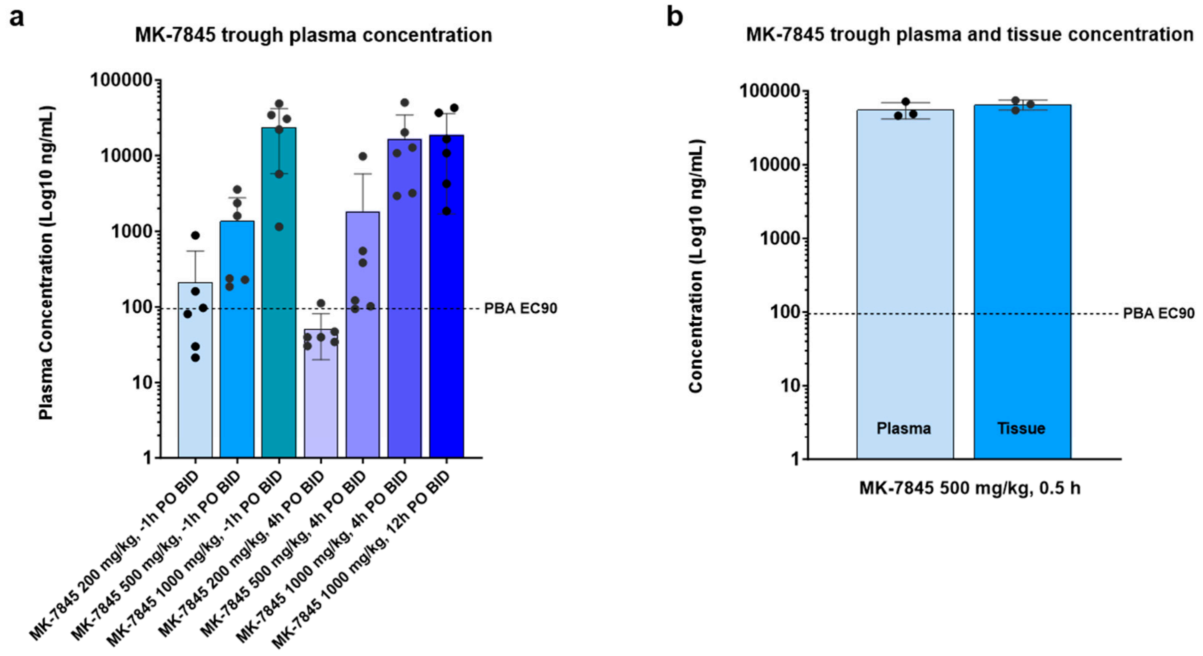

Figure S7 MK-7845 trough levels in MERS-CoV model. **a**) Trough plasma concentration of MK-7845 collected 12 h after the final dose in MERS-CoV infected mice; **b**) Trough plasma and tissue concentrations of MK-7845 at 0.5 h after a single oral dose of 500 mg/kg in CD-1 mice. Plasma binding adjusted (PBA) A549 Replicon assay EC_{90} is represented by the dashed line.

a

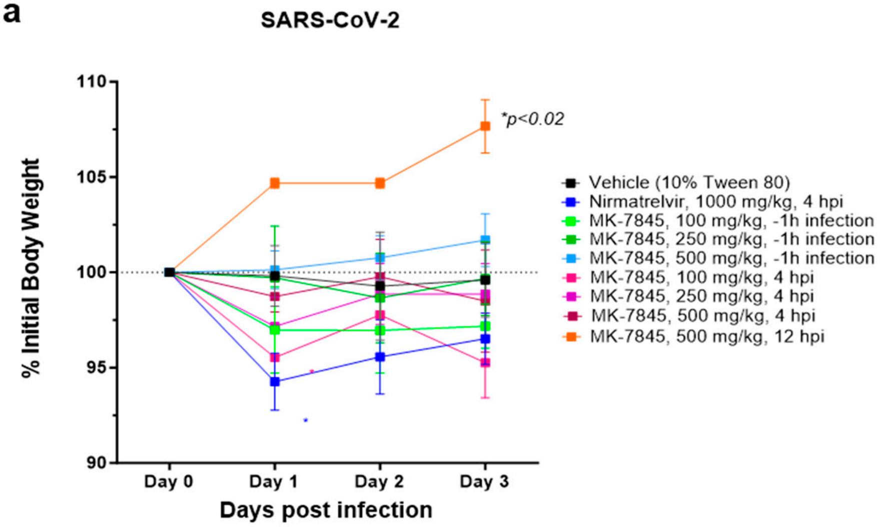

b

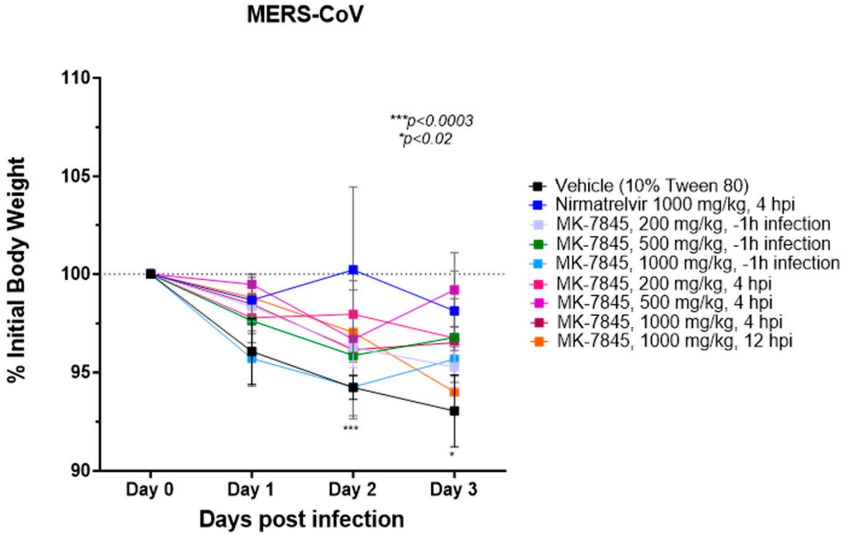

Figure S8 Body weight curves. **a)** K18-hACE2 mice infected with SARS-CoV-2 and **b)** K18-hDPP4 mice infected with MERS-CoV. Body weights of mice intranasally infected and treated with nirmatrelvir, MK-7845 or vehicle-treated (10% Tween 80) were monitored daily from day 0 to 3 dpi. The dotted line indicates 100% of the initial body weight. All body weights were normalized to the percentage of the initial body weight.

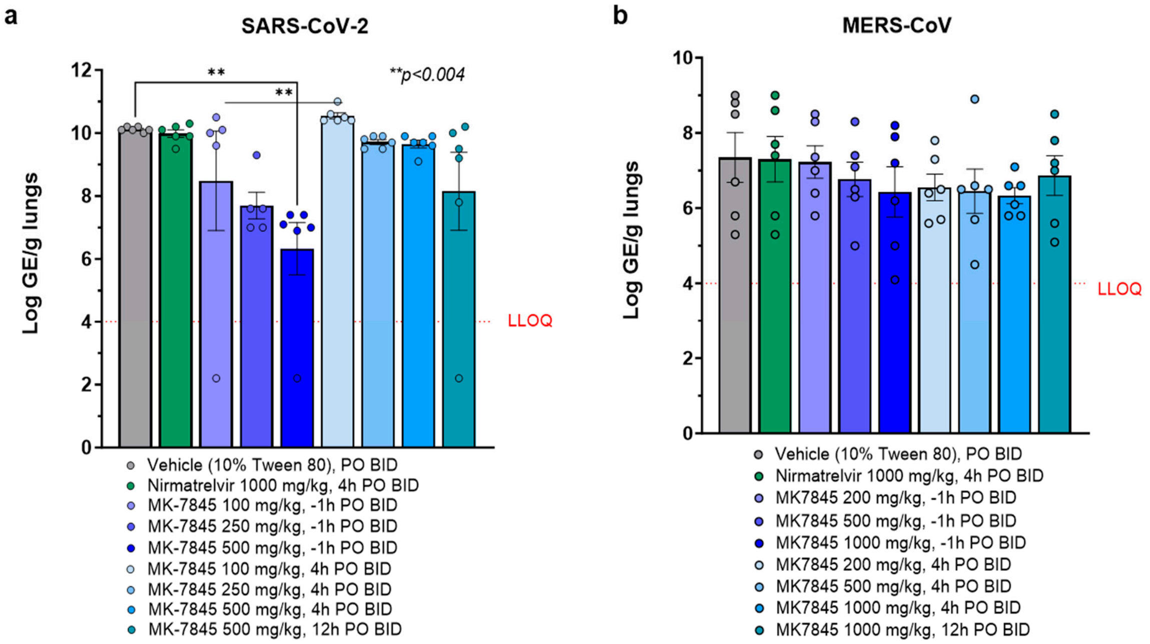

Figure S9 Lung burden analysis by RT-qPCR after intranasal infection. SARS-CoV-2 (**a**) and MERS-CoV (**b**). Graphs show log10 of lung viral genomic RNA equivalent per gram ± SEM. Samples with values below the lower limit of detection (LLOQ) were assigned a value half of LLOQ for analyses. Statistical significance was evaluated by one-way ordinary ANOVA with Dunnett's multiple comparisons test.

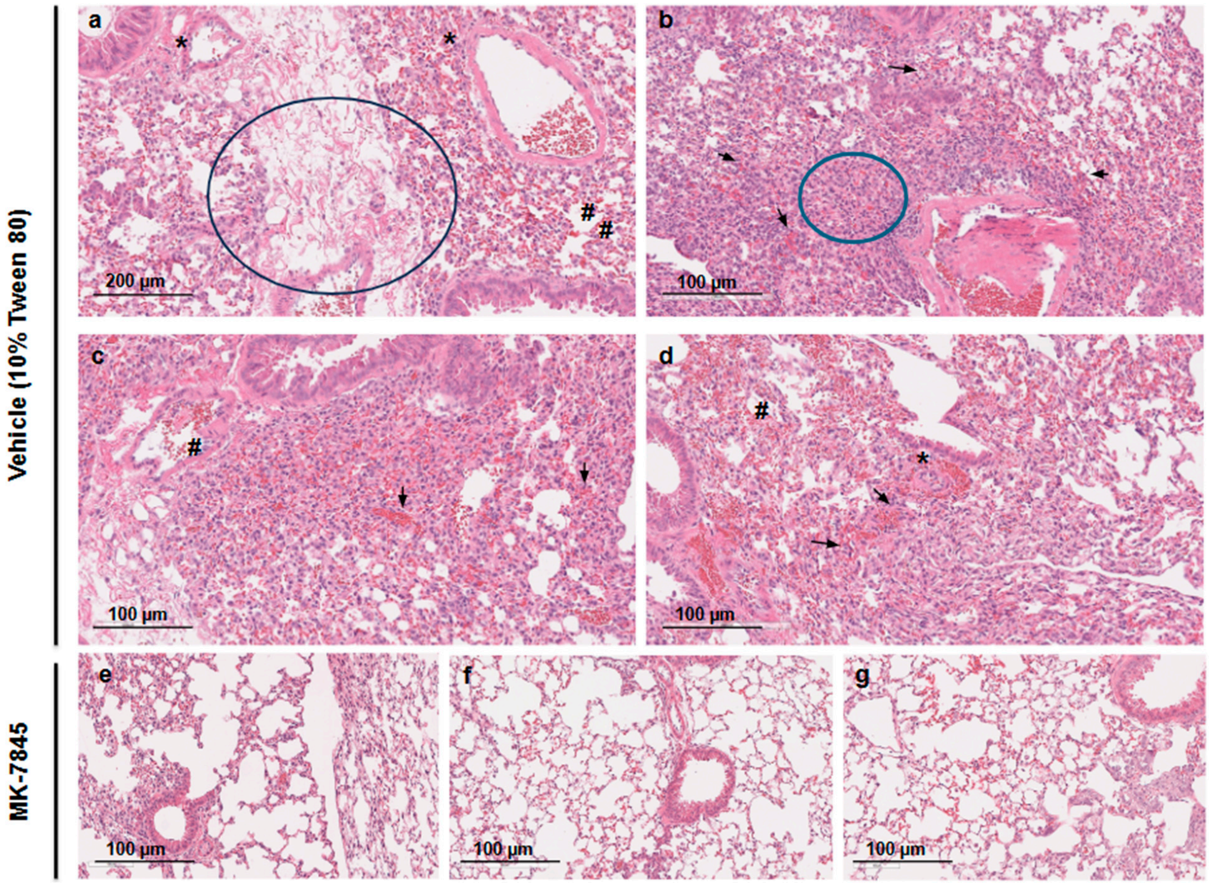

Figure S10 Histopathological analysis of SARS CoV-2 infection in transgenic K18-hACE2 mice treated with MK-7845. Representative images of H&E- stained lung sections from the vehicle-treated group (**a-d**) showing lung damage represented as hemorrhage into alveoli (*arrows*), degeneration of the vascular wall (*asterisk*), perivascular lymphocytes (*hash*), perivascular edema (*black circle*), perivascular inflammation (*blue circle*), and bronchial epithelial cell necrosis, which was protected by the MK-7845 treatment in prophylactic mode at the doses 250 mg/kg (**e**) and 500 mg/kg (**f** and **g**), administered 1 h prior to infection.

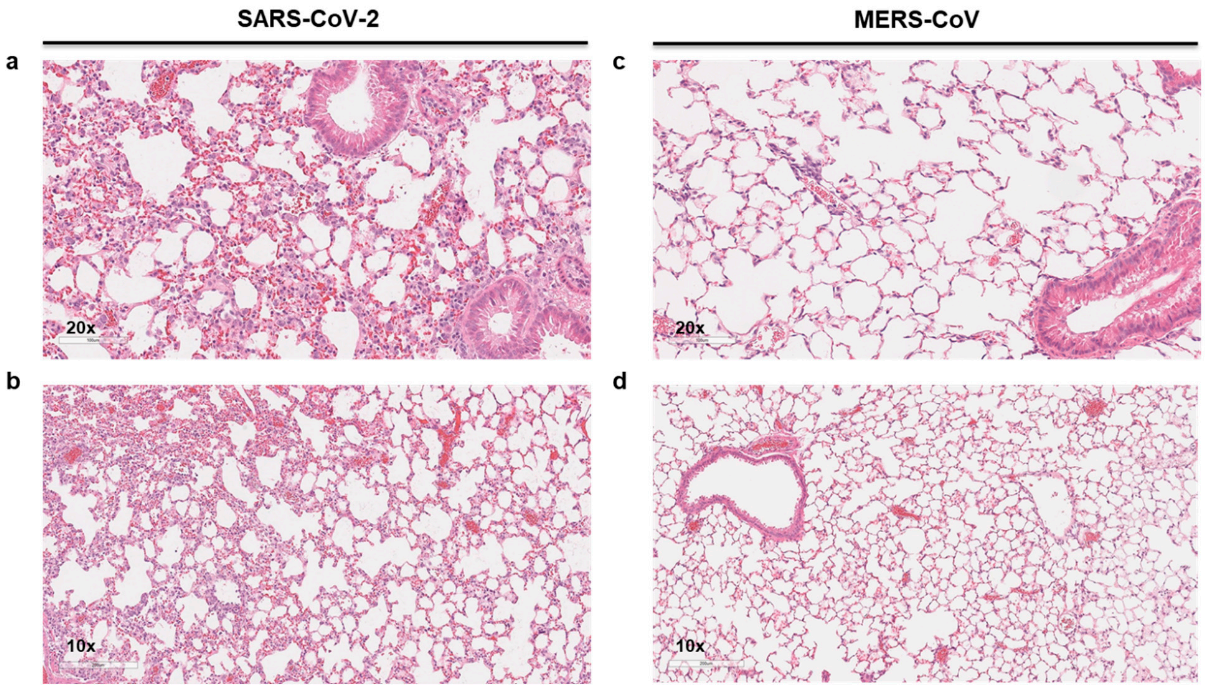

Figure S11 Nirmatrelvir in vivo efficacy against SARS-CoV-2 and MERS-CoV infection. Representative images of H&E- stained lung sections from the nirmatrelvir-treated group infected with SARS-CoV-2 (**a** & **b**) and MERS-CoV (**c** & **d**). Images **a** & **c** are represented at 20x magnification; images **b** & **d** are represented at 10x magnification.

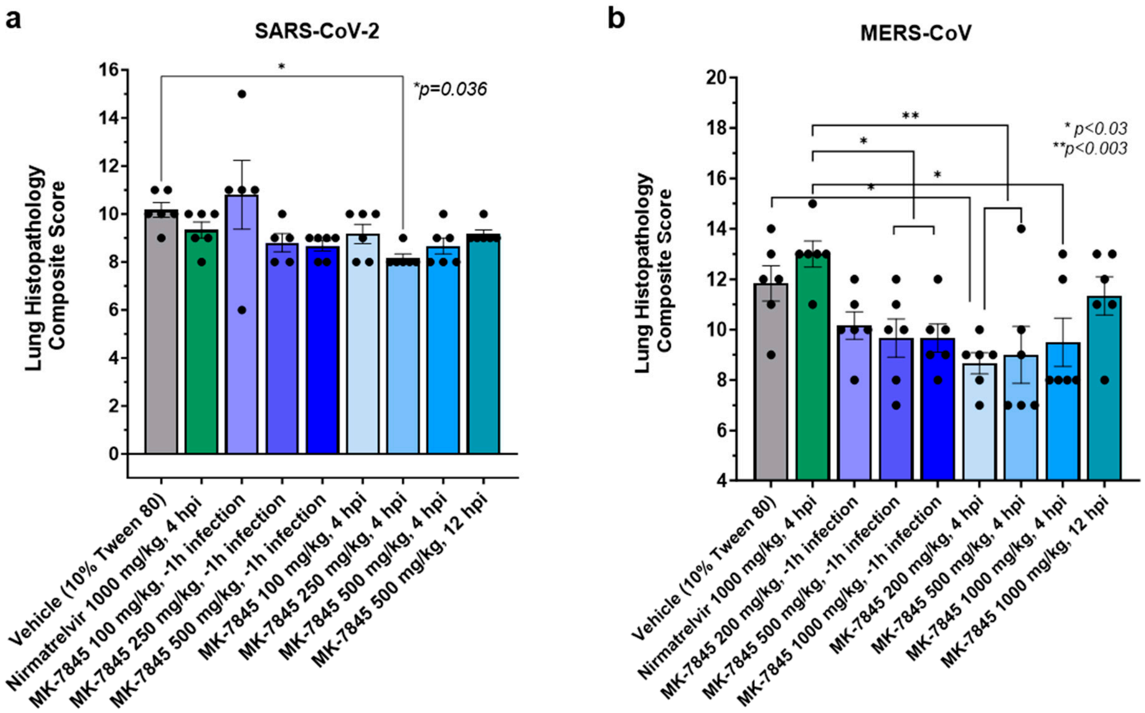

Figure S12 Lung histopathology composite score. Semi-quantitative analysis of histopathological changes in H&E- stained mice lungs after SARS-CoV-2 (**a**) and MERS-CoV infection (**b**).

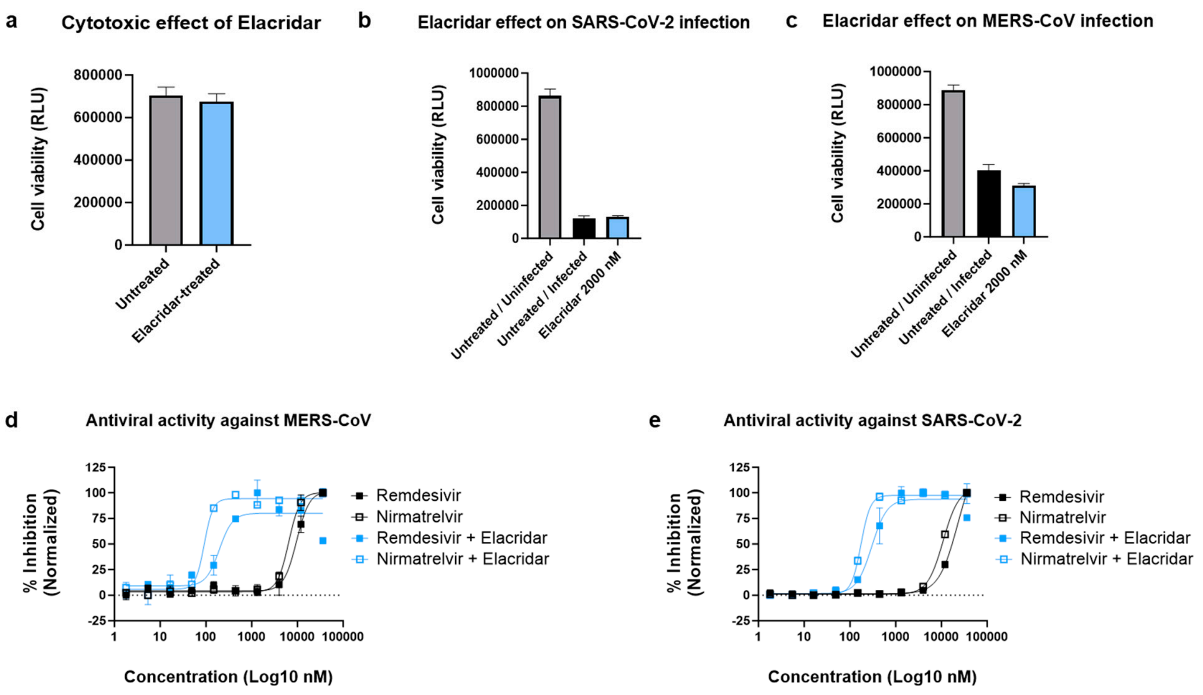

Figure S13 P-GP inhibitor evaluation of activity and cytotoxicity in Vero E6+TMPRSS2 cells. Effect of Elacridar in **a**) cells viability after 72 h incubation; **b**) infection with SARS-CoV-2 (Omicron EG.5.1); **c**) infection with MERS-CoV; **d**) remdesivir and nirmatrelvir activity against SARS-CoV-2 (Omicron EG.5.1); and **e**) remdesivir and nirmatrelvir activity against MERS-CoV.

Table S1 GISAID EPI_SET_240702sw Additional Information

**Data Availability**
GISAID Identifier: EPI_SET_240702sw<br>doi: 10.55876/gis8.240702sw<br>All genome sequences and associated metadata in this dataset are published in GISAID's EpiCoV database. To view the contributors of each individual sequence with details such as accession number, Virus name, Collection date, Originating Lab, Submitting Lab and the list of Authors, visit 10.55876/gis8.240702sw
**Data Snapshot**
EPI_SET_240702sw is composed of 182 individual genome sequences.<br>The collection dates range from 2019-12-31 to 2024-05-21.<br>Data was collected in 43 countries and territories.<br>All sequences in this dataset are compared relative to hCoV-19/Wuhan/WIV04/2019 (WIV04), the official reference sequence employed by GISAID (EPI_ISL_402124). Learn more at https://gisaid.org/WIV04.

Table S2 Sequence of Primers and Probe used for SARS CoV-2 (a) and MERS-CoV (b).

a. SARS-CoV-2			
**Name**	**Description**	**Sequence (5'>3')**	**Label**
E_Sarbeco_F	E gene forward primer	ACAGGTACGTTAATAGTTAATAGCGT	n/a
E_Sarbeco_R	E gene reverse primer	ATATTGCAGCAGTACGCACACA	n/a
E_Sarbeco_Probe	E gene probe	FAM-ACACTAGCCATCCTTACTGCGCTTCG-BHQ-1	FAM+BHQ-1
RP-F	RNAse P Forward Primer	AGA TTT GGA CCT GCG AGC G	n/a
RP-R	RNAse P Reverse Primer	GAG CGG CTG TCT CCA CAA GT	n/a
RP-P	RNAse P Probe	FAM – TTC TGA CCT GAA GGC TCT GCG CG – BHQ-1	FAM+BHQ-1

b. MERS-CoV			
**Name**	**Description**	**Sequence (5'>3')**	**Label**
MERS ORF1a_F	MERS ORF1a gene forward primer	CCACTACTCCCATTTCGTCAG	N/A
MERS ORF1a_R	MERS ORF1a gene reverse primer	CAGTATGTGTAGTGCGCATATAAGCA	N/A
MERS ORF1a Probe	MERS ORF1a probe	FAM-TTGCAAATTGGCTTGCCCCCACT-BHQ-1	FAM+BHQ-1
RP-F	RNAse P Forward Primer	AGA TTT GGA CCT GCG AGC G	N/A
RP-R	RNAse P Reverse Primer	GAG CGG CTG TCT CCA CAA GT	N/A
RP-P	RNAse P Probe	FAM – TTC TGA CCT GAA GGC TCT GCG CG – BHQ-1	FAM+BHQ-1

Table S3 Histopathological analysis of the lungs of K18-hACE2 mice infected with SARS-CoV-2. Values are represented as an average of n = 6 per group. Severity Scores: 0) Normal / absent; 1) Localized, 1 - 33% of lung fields, mild; 2) Multifocal, 34 - 66% of lung fields, moderate; 3) > 67% of lung fields, marked.

Group name	Peribronchial inflammation	Perivascular inflammation	Interstitial inflammation	Intra-alveolar inflammation	Bronchial epithelial cell necrosis	Fibrinoid degeneration vascular wall	Composite score	Significance Vehicle vs Test group (One-way ANOVA)	Significance Nirmatrelvir vs Test group (One-way ANOVA)
**Controls**									
Vehicle 4 hpi	2.0	2.0	2.0	2.0	1.3	0.8	10.2		
Nirmatrelvir 1000 mg/kg PO BID 4 hpi	1.8	1.8	2.0	2.0	0.8	0.8	9.3	0.759	
**PO BID -1h infection**									
MK-7845 100 mg/kg	2.0	2.0	2.2	2.2	1.6	0.8	10.8	0.938	0.239
MK-7845 250 mg/kg	2.0	2.0	2.0	2.0	0.6	0.2	8.8	0.305	0.975
MK-7845 500 mg/kg	2.0	2.0	2.0	2.0	0.5	0.2	8.7	0.180	0.901
**PO BID 4 hpi**									
MK-7845 100 mg/kg	2.0	2.0	2.0	2.0	1.2	0.0	9.2	0.586	0.999
MK-7845 250 mg/kg	2.0	2.0	2.0	2.0	0.2	0.0	8.2	0.036*	0.420
MK-7845 500 mg/kg	2.0	2.0	2.0	2.0	0.7	0.0	8.7	0.180	0.901
**PO BID 12 hpi**									
MK-7845 500 mg/kg	2.0	2.0	2.0	2.0	1.0	0.2	9.2	0.586	0.999

Table S4 Histopathological analysis of the lungs of K18-hDPP4 mice infected with MERS-CoV. Values are represented as an average of n = 6 per group. Severity Scores for alveolar edema, hemorrhage, and consolidation: 1) Normal / absent; 2) Localized, 1 - 33% of lung fields; 3) multifocal, 34 - 66% of lung fields; 4) common, > 67% of lung fields. Severity scores for cell debris in lymphatics and vascular thrombi: 1) normal / absent; 2) < 1 per 100 x field; 3) 1 - 2 per 100 x fields; 4) > 2 per 100 x fields. Some samples showed perivascular lymphocyte infiltration (PVLI) in the vehicle-treated group, MK-7845 at 200 mg/kg and 500 mg/kg 1 h prior to infection, as well as in 200 mg/kg and 1000 mg/kg at 4 hpi and 1000 mg/kg at 12 hpi (*data not shown*).

Group name	Alveolar Edema	Hemorrhage	Consolidation	Cell debris - Lymphatics	Vascular Thrombi	Composite score	Significance Vehicle vs Test group (One-way ANOVA)	Significance Nirmatrelvir vs Test group (One-way ANOVA)
**Controls**								
Vehicle 4 hpi	2.8	2.7	2.8	2.2	3.2	11.8		
Nirmatrelvir 1000 mg/kg PO BID 4 hpi	2.0	2.5	3.0	1.7	1.8	13.0	0.815	
**PO BID -1h infection**								
MK-7845 200 mg/kg	1.8	2.8	3.0	2.5	2.0	10.2	0.479	0.055
MK-7845 500 mg/kg	1.3	2.5	3.0	1.5	2.2	9.7	0.216	0.016
MK-7845 1000 mg/kg	1.7	2.7	3.0	2.0	2.5	9.7	0.216	0.016
**PO BID 4 hpi**								
MK-7845 200 mg/kg	1.7	2.3	2.7	1.5	1.0	8.7	0.025	0.001
MK-7845 500 mg/kg	1.8	2.0	2.7	1.5	1.0	9.0	0.055	0.003
MK-7845 1000 mg/kg	1.3	2.3	2.2	1.5	1.0	9.5	0.158	0.011
**PO BID 12 hpi**								
MK-7845 1000 mg/kg	1.3	2.0	2.3	1.2	1.0	11.3	0.997	0.479
